# Organocatalytic Enantioselective α-Bromination of Aldehydes with *N*-Bromosuccinimide

**DOI:** 10.1021/acs.joc.2c00600

**Published:** 2022-05-26

**Authors:** George Hutchinson, Carla Alamillo-Ferrer, Martín Fernández-Pascual, Jordi Burés

**Affiliations:** The University of Manchester, Department of Chemistry, Oxford Road, M13 9PL Manchester, U.K.

## Abstract

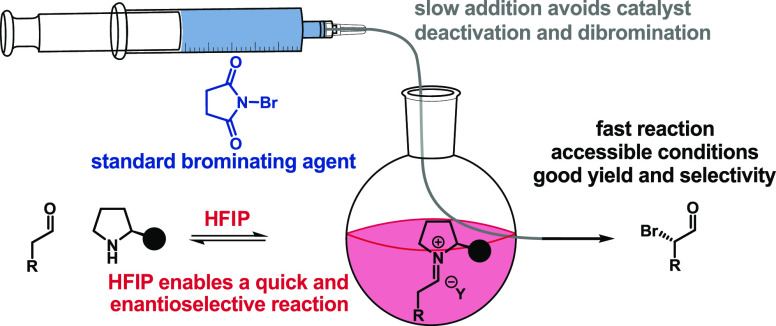

Despite the wealth of existing organocatalytic, enantioselective transformations, the α-bromination of aldehydes remains a challenging reaction. The four examples reported to date require expensive, inconvenient brominating agents to achieve the desired products in excellent yields and enantioselectivities. The preferred brominating agent, *N*-bromosuccinimide (NBS), has been repeatedly discarded for these reactions because it results in low yields and relatively poor enantioselectivities. We describe a methodology that uses NBS and performs excellently with low catalyst loadings, short reaction times, and mild temperatures.

## Introduction

**Figure 1:**
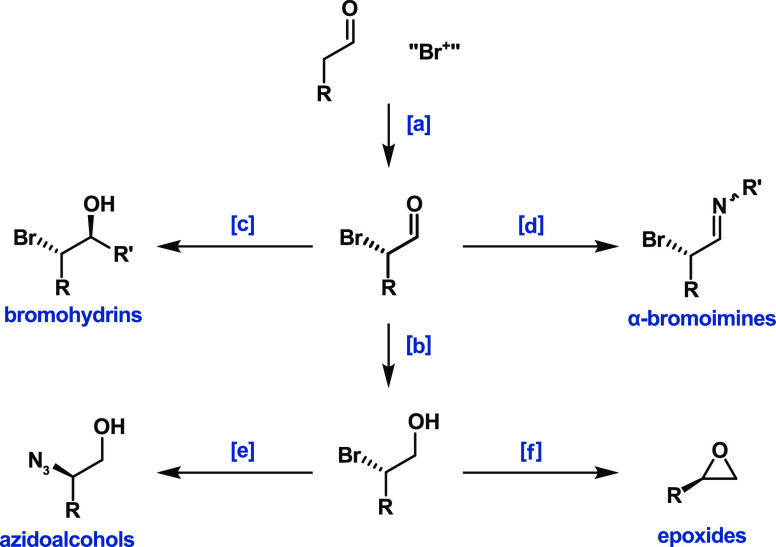
α-Brominated aldehydes are versatile synthons. [a] Any of the previous four methods^4−7^ or this work. [b] NaBH_4_, MeOH.^4−7^ [c] R’MgBr.^6^ [d] R’NH_2_, MgSO_4_.^8a^ [e] (1) Alcohol protection; (2) NaN_3_;^8b^ and (3) alcohol deprotection. [f] NaH.^8c^

Over the past 20 years, asymmetric organocatalysis has emerged as a potent strategy for the formation of a diverse range of useful molecules.^1^ This revolutionary work culminated in the 2021 Nobel Prize in Chemistry being awarded to List and MacMillan for their pioneering work. Chiral secondary amines have been used as catalysts in a wealth of stereoselective transformations, such as the installation of heteroatoms at the α-position of aldehydes. While procedures for the enantioselective, organocatalytic fluorination^2^ or chlorination^3^ of aldehydes have been widely reported, only a few α-brominations of aldehydes have been described.^4−7^ These α-bromination reactions provide highly versatile chiral building blocks that can be rapidly derivatized to α-bromoimines,^8a^ azidoalcohols,^8b^ epoxides,^8c^ or bromohydrins (Figure 1).^4−7^

**Figure 2:**
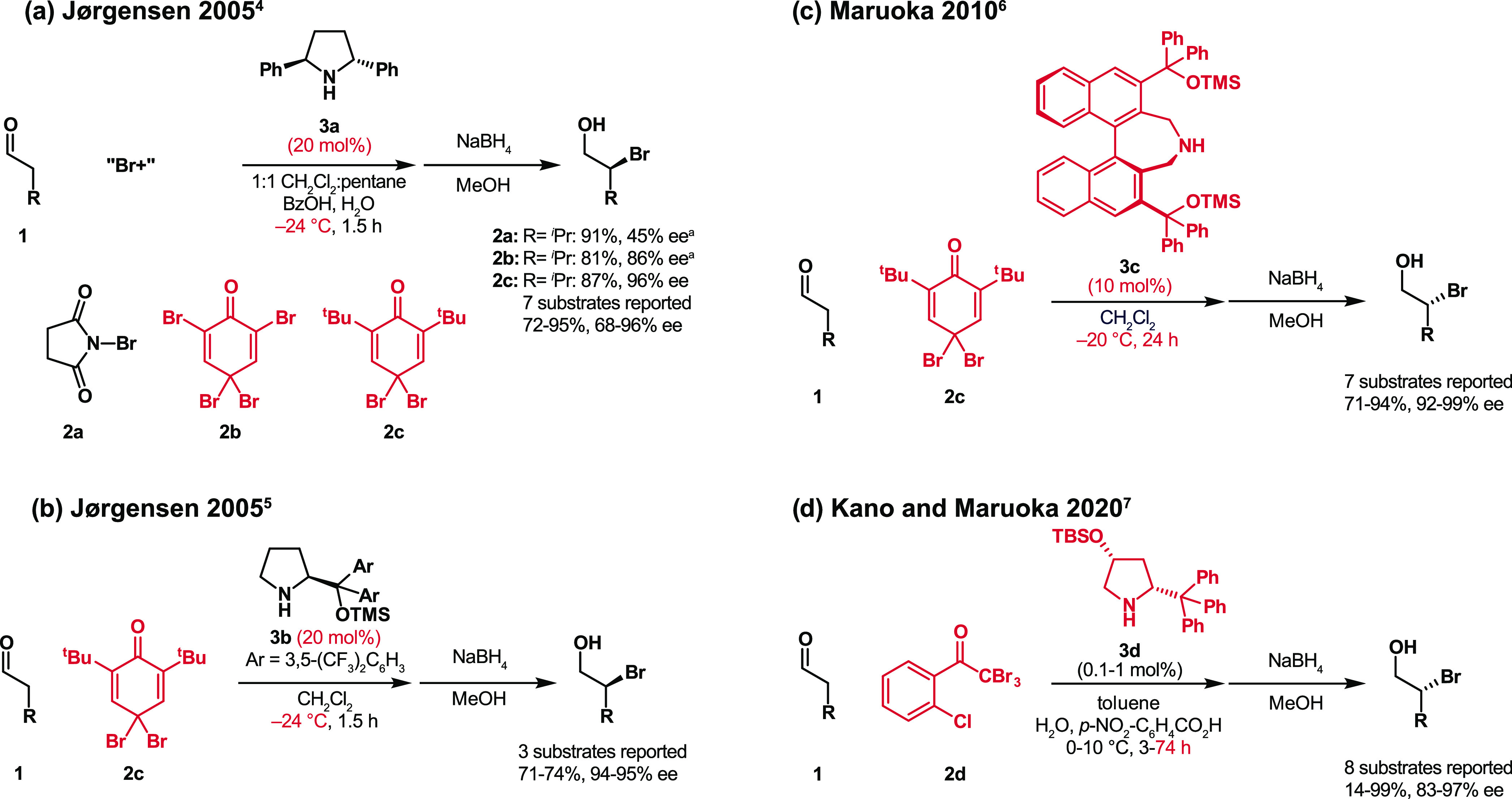
Previous examples of enantioselective aminocatalytic α-brominations of aldehydes. Nonideal conditions and reactants are highlighted in red. ^a^1:3 pentane:CH_2_Cl_2_ and no added water.

The first organocatalytic, enantioselective α-bromination of aldehydes was described by the Jørgensen group in 2005 (Figure 2a).^4^ The authors began their investigation using the reaction conditions from their successful chlorination but used *N*-bromosuccinimide (NBS, **2a**) instead of *N*-chlorosuccinimide (NCS). The group found that NBS was an unsuitable brominating agent under these reaction conditions, giving just 8% conversion and 19% enantiomeric excess (Figure 2a). The authors attributed these poor results to the “increased reactivity of NBS **2a** compared to that of NCS.” Hence, they had to resort to using 4,4-dibromo-2,6-di-*tert*-butyl-cyclohexa-2,5-dienone (**2c**) as the brominating agent to achieve excellent yields and enantioselectivities. The main handicap of this methodology is the necessity to use the unusual brominating agent **2c**, which up to date, has only been used in 48 reactions, whereas the ubiquitous NBS has been used in 4,838,258 reactions.^9^

Later in the same year (Figure 2b),^5^ the Jørgensen group reported an improved methodology that used a more convenient catalyst, (*S*)-α,α-bis[3,5-bis(trifluoromethyl)phenyl]-2-pyrrolidinemethanol trimethylsilyl ether (**3b**) but still required the use of the unusual brominating agent, 4,4-dibromo-2,6-di-*tert*-butyl-cyclohexa-2,5-dienone **2c**, instead of NBS. As in their previous methodology, the authors still required low temperatures (−24 °C) to prevent side reactions, such as bromination of the catalyst.

Maruoka et al. described another example of an aminocatalytic, enantioselective bromination in 2010 (Figure 2c).^6^ The authors tested seven potential brominating agents and observed that five of them, most notably NBS (**2a**), gave no conversion (<5%). Therefore, the authors had to use the same nonideal brominating agent as Jørgensen, **2c**. They used 10 mol % of the Maruoka’s binaphthyl catalyst **3c**, which provides the best-reported enantioselectivities but is very laborious to synthesize.^6,10^

In 2020, Maruoka and Kano explored alternative aminocatalytic methods using pyrrolidine derivatives instead of **3c** as catalyst (Figure 2d).^7^ They reported the rapid decomposition of NBS (**2a**) and 4,4-dibromo-2,6-di-*tert*-butyl-cyclohexa-2,5-dienone (**2c**) in the presence of pyrrolidine and explored milder brominating agents to avoid the bromination of the pyrrolidine derivatives used as catalysts. They explored several noncommercially available ketone-based brominating agents (KBAs) and chose to use ClPh-KBA **2d** (Figure 2d). Even using KBAs, the standard Jørgensen–Hayashi catalyst provided poor yields and enantioselectivities, making necessary the use of noncommercially available pyrrolidine-based catalysts with very large substituents in positions 2 and 4 (e.g., **3d**). This original combination of catalyst and brominating agent reduced the catalyst deactivation significantly, allowing the authors to obtain excellent yields even with only 0.1 mol % of catalyst in 74 h.

In brief, both groups pioneering the enantioselective aminocatalytic α-bromination of aldehydes, Jørgensen’s and Maruoka’s, discarded the possibility of using the commonly preferred brominating agent, NBS. Their original solutions had to compromise on the brominating agent and reaction conditions to get excellent yields and enantioselectivities. All of the brominating agents reported by Jørgensen and Maruoka generate stochiometric amounts of organobromine byproducts, which are toxic and environmental hazards.^11^ Additionally, due to the inherent difficulty of the transformation, the turnover frequencies of their methodologies are generally low, hence requiring either very high catalyst loadings (up to 20 mol %)^4,5^ or very long reaction times (74 h when using 0.1 mol % of catalyst).^7^ Some of the reactions also involve atypical, noncommercially available catalysts and low temperatures (lower than −20 °C). Herein, we describe a method that achieves excellent turnover frequencies and enantioselectivities using NBS and 2 mol % of a Jørgensen–Hayashi type catalyst at convenient temperatures.

We have recently described an aminocatalytic, enantioselective α-chlorination of aldehydes using 1,1,1,3,3,3-hexafluoroisopropanol (HFIP)^12^ as the solvent.^3f^ We chose this solvent with the intention of shifting the reaction mechanism to proceed through charged intermediates rather than stable, neutral species. This strategy allowed us to chlorinate a range of aldehydes with good yields and excellent enantioselectivities, using low catalyst loadings, commercially available reagents, short reaction times, and convenient temperatures. We anticipated that the application of a similar knowledge-based strategy would yield comparable improvements for an enantioselective α-bromination of aldehydes.

## Results and Discussion

Although we used our method for the aminocatalytic chlorination in HFIP as starting point, the higher reactivity of NBS with respect to NCS made the development of the bromination methodology much more challenging than the chlorination one. A better control of the reaction conditions is necessary to avoid the deactivation of the aminocatalyst, which occurs quicker with NBS than NCS. The reaction conditions have to be modified to minimize the undesired dihalogenation of the aldehyde, more prevalent in the bromination than the chlorination. The reaction time has to be precisely controlled to avoid the racemization of the α-brominated aldehydes, which are less stable than the α-chlorinated. Also, the optimization of the reaction conditions could not be guided by in situ FTIR because the IR probe is chemically incompatible with the reaction media.^13^

**Figure 3:**
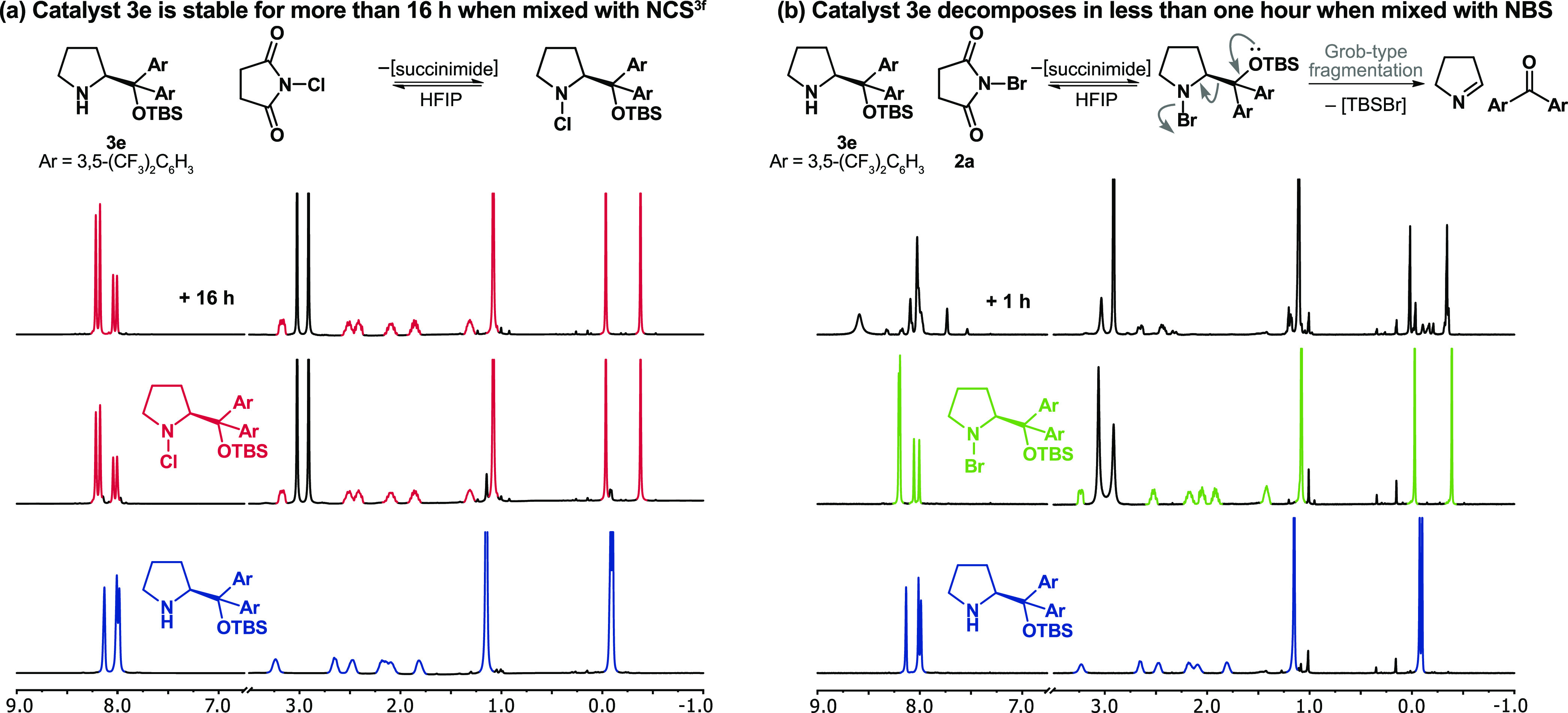
Jørgensen–Hayashi catalyst is far less stable when brominated than when chlorinated.

First, we compared the stability of the Jørgensen–Hayashi type catalysts when mixed with NBS or NCS in HFIP. The OTBS–prolinol derivative bearing 3,5-bis(trifluoromethyl)phenyl groups (**3e**), which is stable for more than 16 h when chlorinated (Figure 3a), decomposes over just 1 h after being brominated (Figure 3b). The faster irreversible deactivation of the halogenated aminocatalyst meant that it was more challenging and more important to control the undesired reaction of the catalyst with the halogenating agent during the bromination than it had been during the chlorination.

A further complication of the bromination reaction is the lower stability of the product of the reaction, the α-brominated aldehyde, compared with the chlorinated analogue. We have observed that the monobrominated aldehyde loses enantiomeric excess not only when mixed with the aminocatalyst in HFIP but also when mixed with the apparently innocuous byproduct of the reaction, succinimide.^14^ Maruoka et al. also described the high instability of brominated products when they noticed the loss of enantiomeric excess during NaBH_4_ reduction.^6^

**Figure 4:**
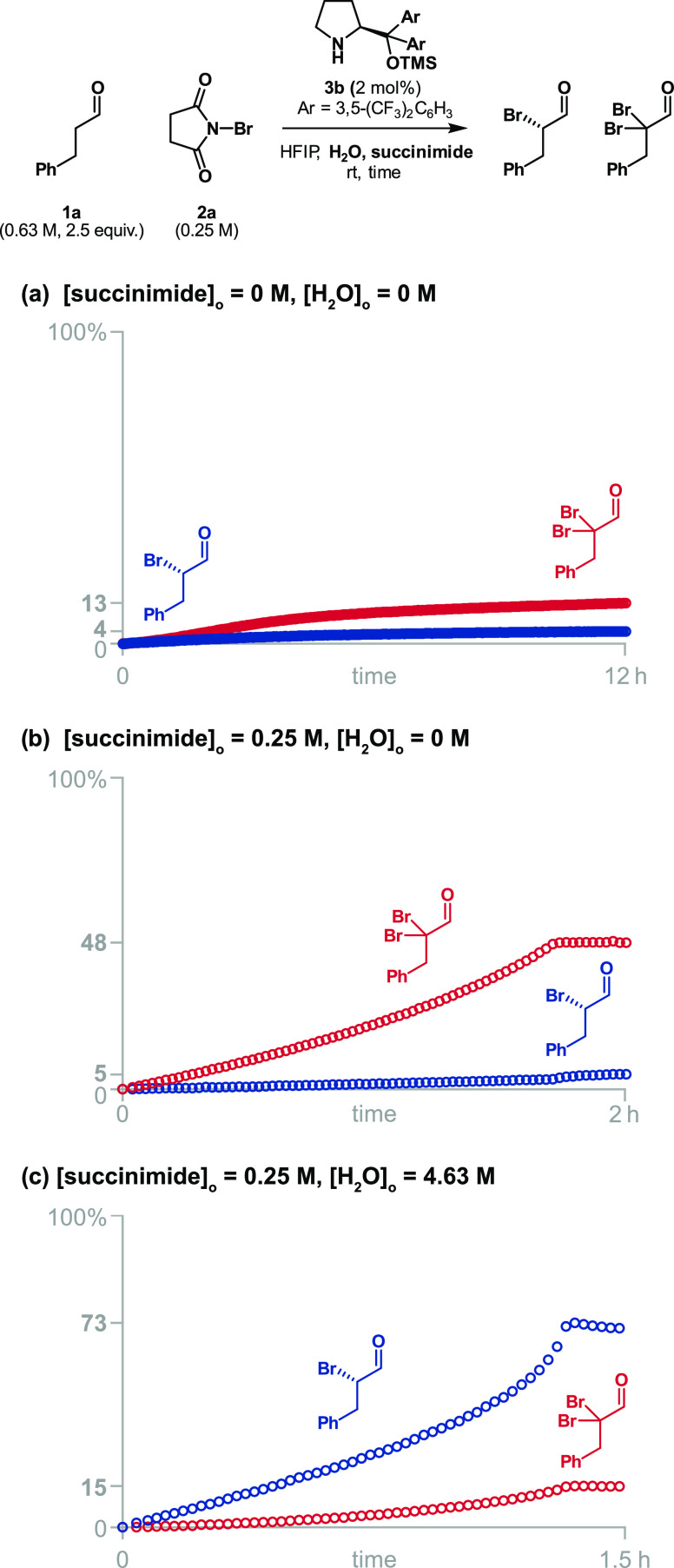
Adding succinimide enhanced the reaction rate, while adding water reduced the dibromination, as demonstrated by monitoring the reaction by ^1^H NMR.

When we tested the aminocatalytic reaction with 2 mol % of the Jørgensen–Hayashi catalyst **3f** and NBS under standard conditions in HFIP, we obtained mostly dibrominated aldehyde (13%) with only some of the desired product (4%) after 12 h (Figure 4a). This result is consistent with the yields reported by Jørgensen (8%) and Maruoka (<5%) and confirms the difficulty of using NBS in this transformation. We observed, by NMR, that most of the catalyst was brominated in the first 5 min after starting the reaction, which explains the low yields. We attempted to mitigate the catalyst deactivation by adding succinimide to the reaction mixture. The addition of succinimide shifts the equilibrium between free and brominated catalyst toward free catalyst and, hence, accelerates the main reaction. In the presence of succinimide, 2 mol % of **3f** was sufficient to consume all of the NBS, but the dibrominated aldehyde was still the main product of the reaction (Figure 4b). The large percentage of dibrominated aldehyde arose because of the over bromination of the brominated enamine, one of the key reaction intermediates of the catalytic reaction, instead of its hydrolysis. In this bifurcation, the branching ratio of products is affected by the concentration of reactants involved in each pathway and therefore water should reduce the percentage of dibromination. Indeed, the addition of water increased the yield of product from 5 to 73% (Figure 4c) and reduced the formation of dibrominated aldehyde from 48 to 15% (Figure 4c), but the enantiomeric ratio of the product was only 78:22. While the additions of succinimide and water to reactions using NBS mitigated the catalyst deactivation and aldehyde dibromination, the enantioselectivity was unsatisfactory. This low enantioselectivity is likely due to the aforementioned erosion of product’s enantiomeric excess in the presence of succinimide.

To increase the performance of the aminocatalytic α-bromination of aldehydes using NBS, we explored the slow addition of the brominating agent to the reaction mixture. The slow addition maintains a low concentration of NBS in the reaction media, which reduces the dibromination and favors the irreversible bromination of the aldehyde over the reversible bromination of the catalyst. For each substrate, we increased the time of addition of NBS until we observed full consumption at the end of the addition, which guarantees that the NBS does not build up at any point of the reaction. In addition, we quenched the reaction at the end of the addition of NBS to minimize product racemization.

The amount of water allows us to tune the ratio between the product and the dibrominated aldehyde. Previous studies of the chlorination and fluorination reactions suggest the second halogenation acts as a kinetic resolution, enantioenriching the product of the reaction.^3f,5^ However, in the bromination reaction, the correlation between the percentage of dibromination and enantiomeric excess of the product is much smaller. This is especially the case for short-chain aldehydes,^14^ which are challenging substrates absent from the substrate scope of the previous methodologies.^4−7^

By tuning the time of addition and amount of water, we were able to use NBS for the aminocatalytic α-bromination of aldehydes without sacrificing yield or enantioselectivity. We also achieved great turnover frequencies, which allowed us to run most of the reactions in less than 1.5 h using only 2 mol % of the Jørgensen–Hayashi type catalyst **3e**. Some substrates, particularly hydrocinnamaldehyde (Table 1, entry 1) and octanal (Table 1, entry 3), performed exquisitely under our reaction conditions, giving good yields and high enantioselectivities after very quick optimization of the reaction conditions. We successfully increased the scale of the bromination of hydrocinnamaldehyde while maintaining a good yield at the cost of some enantiomeric excess (Table 1, entry 2). We found that dodecanal displayed a tendency to dibrominate, giving a lower yield but comparable enantiomeric excess to octanal with the same amount of added water (Table 1, entry 4). For pentanal, a shorter chain substrate, we attained a good yield after increasing the amount of water and time of addition (Table 1, entry 5). The α-bromination of propanal yielded moderate enantioselectivities (Table 1, entry 6), but the result is remarkable given that it is the first reported enantioselective bromination of the shortest utilizable linear aldehyde. We obtained excellent enantioselectivities for isovaleraldehyde, which required longer addition times (4.75 h) because it is β-branched (Table 1, entry 7). Similarly, we found that the bromination of 3-cyclohexylpropanal had to be carried out at room temperature to allow full consumption of the NBS over a reasonable amount of time (Table 1, entry 8).

**Table 1:**
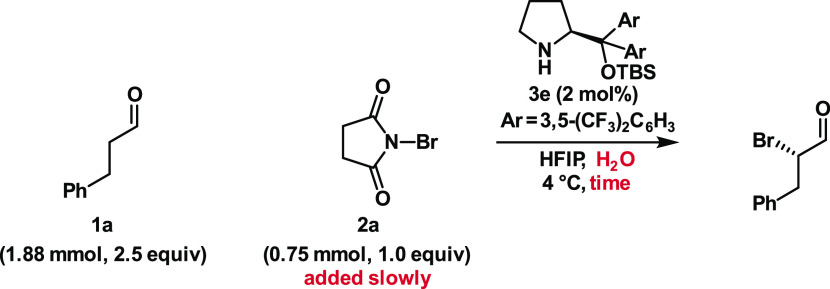
Examples of Optimized Reaction Conditions for the α-Bromination of Aldehydes

entry	deviation from above	H_2_O (μL)	time of addition of NBS (min)	yield^a^ (%)	er^b^
1	none	50	60	71 (65)	98:2
2	**3 mmol scale** (4x) instead of 0.75 mmol	200	60	72 (58)	92:8
3	**octanal** instead of hydrocinnamaldehyde	80	60	73 (59)	95:5
4	**dodecanal** instead of hydrocinnamaldehyde	80	60	44 (40)	96:4
5	**pentanal** instead of hydrocinnamaldehyde	100	75	65 (47)	92:8
6	**propanal** instead of hydrocinnamaldehyde	200	150	61 (33^c^)	76:24
7	**isovaleraldehyde** instead of hydrocinnamaldehyde	85	285	69 (51)	95:5
8^d^	**3-cyclohexylpropanal** instead of hydrocinnamaldehyde	50	90	72 (62)	90:10

a Yield of α-bromoaldehyde measured by qNMR with an internal standard (see Electronic Supporting Information (ESI)) after reduction of the α-bromoaldehyde to the bromohydrin. The number in parenthesis indicates the isolated yield of the bromohydrin after purification from a repeated reaction.

b Enantiomeric ratio determined by chiral high-performance liquid chromatography (HPLC) after reduction of the α-bromoaldehyde to the bromohydrin. Entries 3–8 were benzoylated for UV detection during HPLC analysis.

c Product was volatile and was isolated after benzoylation of the bromohydrin.

d Reaction run at room temperature.

## Conclusions

In conclusion, the organocatalytic, enantioselective α-bromination of aldehydes is a much more challenging reaction than the analogous chlorination because of the greater reactivity of the brominating agents and instability of the products. We have overcome the limitations of previous methods with respect to the use of NBS as a brominating agent using HFIP as a solvent and tuning the amount of water and dosing the NBS during the reaction. The use of NBS is a more environmentally friendly alternative to the previous brominating agents because it avoids the stoichiometric formation of organobromine byproducts. Our methodology does not require special catalysts or lower temperatures to minimize the catalyst deactivation, which allows us to achieve, in most cases, better turnover frequencies than the previous methodologies.

## Experimental Section

### General Information

Commercially available aldehydes were carefully distilled under vacuum into an LN_2_ trap immediately prior to use. The 3-cyclohexylpropanal was synthesized by Dess–Martin periodinane (DMP) oxidation of 3-cyclohexylpropanol, following GP1. The *N*-bromosuccinimide (NBS) was recrystallized from water. The (*S*)-α,α-bis[3,5-bis(trifluoromethyl)-phenyl]-2-pyrrolidinemethanol trimethylsilyl ether (**3c**) and (*S*) -α,α-bis[3,5-bis(trifluoromethyl)-phenyl]-2-pyrrolidinemethanol *tert*-butyldimethylsilyl ether (**3e**) catalysts were purified from commercial sources by flash column chromatography (CH_2_Cl_2_) to remove any deprotected alcohol. All other reagents and solvents were used as-purchased from Merck, Fluorochem, Alfa Aesar, and TCI. All NMR spectra were recorded on a Bruker AVII 500 MHz spectrometer or a Bruker AVIII HD 400 MHz spectrometer with BBO prodigy probe. ^1^H NMR and ^13^C NMR chemical shifts (δ) are quoted in ppm relative to residual solvent peaks (for ^1^H and ^13^C, respectively, given in ppm, for CDCl_3_: 7.26, 77.16). Any nondeuterated NMR spectra were recorded after shimming on the solvent peak closest to the middle of the spectrum and are reported with respect to the shift of this solvent peak aligned with its position in CDCl_3_ (for ^1^H and ^13^C, respectively, given in ppm, for HFIP: 4.49, 69.20). Chiral HPLC was carried out on an Agilent 1260 Infinity II LC equipped with a diode array detector. Slow additions were carried out using a Harvard Apparatus standard infuse/withdraw pump 11 elite programmable syringe pump calibrated to the syringe, a Henke-Saas-Wolf Air-tight 2.5 mL. The brominating agent was added as a stock solution through poly(tetrafluoroethylene) (PTFE) tubing with an internal diameter of 0.50 mm. All bromination reactions were carried out in a STEM Integrity 10 set to the desired temperature. Flash column chromatography was performed using 230–400 mesh silica, with the indicated solvent system according to standard techniques. Analytical thin-layer chromatography (TLC) and preparative thin-layer chromatography were performed on precoated glass-backed silica gel plates (Supelco TLC Silica gel 60 F_254_). Visualization of the developed chromatogram was performed by UV absorbance (254 nm) or anisaldehyde stain. The time of addition and amount of water required for each substrate were optimized following the procedure described for chlorination in our previous work.^3f^ Yields were calculated after reduction of the α-bromoaldehydes to the corresponding bromohydrins using qNMR with an internal standard (see ESI).

### General Procedure 1 (**GP1**): DMP Oxidation of 3-Cyclohexylpropan-1-ol^15^

To a stirred solution of alcohol (2.0 g, 14 mmol, 1 equiv) in dry CH_2_Cl_2_ (50 mL, 0.28 M) under N_2_ was added DMP (7.2 g, 17 mmol, 1.2 equiv). The reaction mixture was stirred for 3 h before Et_2_O (200 mL) and sat. NaHCO_3(aq)_ (100 mL) was added. After stirring for 10 min, the mixture was filtered through a short plug of celite and transferred to a separating funnel. The organic phase was washed with sat. NaHCO_3(aq)_ (2 × 100 mL) and brine (50 mL) before the organic phase was collected, dried over MgSO_4_, and concentrated under reduced pressure. The crude residue was purified by flash column chromatography (9:1 hexane:EtOAc) to afford a colorless oil (1.70 g, 12 mmol, 86%).

### General Procedure 2 (**GP2**): Bromination of Aldehydes

Solutions of aldehyde (1.88 mmol in 500 μL, 2.5 equiv), (*S*)-α,α-bis[3,5-bis(trifluoromethyl)-phenyl]-2-pyrrolidinemethanol *tert*-butyldimethylsilyl ether **3e** (0.015 mmol in 500 μL, 2 mol %) and H_2_O (determined amount in 500 μL) in HFIP were added to a stirred vial containing HFIP (500 μL) at 4 °C. The reaction mixture was stirred for 2 min before a solution of NBS (0.75 mmol in 1000 μL, 1 equiv) in HFIP was added over the determined time. Immediately after the end of the addition, the reaction mixture was transferred to a stirred flask containing MeOH (1 mL) and CH_2_Cl_2_ (1 mL) before NaBH_4_ (approximately 5 equiv) was added. This mixture was stirred for 2 min before sat. NH_4_Cl_(aq)_ (5 mL), H_2_O (5 mL) and a stock solution of internal standard were added. The mixture was extracted with CH_2_Cl_2_ (4 × 15 mL), before the combined organic phase was washed with brine (15 mL), dried over MgSO_4_, and concentrated under reduced pressure (care should be taken as some of the bromohydrins, particularly 2-bromo-3-methylbutan-1-ol, 2-bromo-pentan-1-ol and 2-bromo-propan-1-ol, are volatile). The products were isolated after purification by flash column chromatography.

### General Procedure 3 (**GP3**): Benzoylation of Alcohols

The crude or purified product from **GP2** was dissolved in dry CH_2_Cl_2_ (10 mL, 0.075 M) before BzCl (3 mmol, 347 μL, 4 equiv) was added. NEt_3_ (3 mmol, 413 μL, 4 equiv) was added dropwise over 30 s. The reaction mixture was stirred overnight before sat. NaHCO_3(aq)_ (10 mL) was added. The mixture was transferred to a separating funnel, and the organic phase was collected, dried over MgSO_4_, and concentrated under reduced pressure. The crude residue was purified by flash column chromatography or preparative TLC.

### General Procedure 4 (**GP4**): Synthesis of Racemic Bromohydrins

To a stirred solution of the aldehyde (3.75 mmol, 1 equiv) in CH_2_Cl_2_ (10 mL, 0.375 M) was added NBS (800 mg, 4.5 mmol, 1.2 equiv) and dl-proline (86 mg, 0.75 mmol, 20 mol %) at room temperature. The reaction mixture was stirred for 2 h before being transferred to a stirred vial containing MeOH (5 mL) and NaBH_4_ (approximately 5 equiv). The reduction was stirred for 2 min before sat. NH_4_Cl_(aq)_ (10 mL) and H_2_O (5 mL) were added. The mixture was extracted with CH_2_Cl_2_ (4 × 30 mL) before the combined organic phase was washed with brine (30 mL), dried over MgSO_4_, and concentrated under reduced pressure.

### Stability of Catalyst **3e** When Brominated

To a stirred solution of (*S*)-α,α-bis[3,5-bis(trifluoromethyl)-phenyl]-2-pyrrolidinemethanol *tert*-butyldimethylsilyl ether (0.06 mmol, **3e**) in HFIP (0.6 mL. 0.1 M) in an NMR tube was added *N*-bromosuccinimide (0.132 mmol, 2.2 equiv). Sequential ^1^H NMR spectra were collected over 90 min.

### Reactions with an Instantaneous Injection of NBS Solution

A stock solution of catalyst **3b** (100 μL of a 0.03 M solution in HFIP, 2 mol %) was added to a mixture of hydrocinnamaldehyde (0.38 mmol, 2.5 equiv), NBS (0.15 mmol), and the desired amounts of succinimide and water in HFIP (500 μL). Sequential ^1^H NMR spectra were collected over the reaction course. The integrals of the aldehyde protons on the starting material (9.72 ppm) and mono- (9.43 ppm) and dibrominated aldehydes (9.26 ppm) were compared to determine the conversion.

### Assessment of the Configurational Stability of the α-Bromoaldehyde Products

2-Bromo-3-phenylpropanal was obtained following GP2 with catalyst **3b**, a 90 min addition of NBS, and 100 μL of added water without NaBH_4_ reduction. The monobrominated aldehyde was purified by column chromatography (100% CH_2_Cl_2_) to give a pale-yellow oil (82 mg, 51% isolated yield, 86:14 er). The isolated monobrominated aldehyde was mixed separately with catalyst **3b** (5 mol %) and with NHS (1.0 equiv). These reactions were sampled and the enantiomeric ratio of the aldehyde in each sample was determined by chiral HPLC after reduction to the corresponding bromohydrin.

#### 2-Bromo-3-phenylpropan-1-ol^7^

2-Bromo-3-phenylpropan-1-ol was obtained following GP2 with an initial amount of 50 μL of water and an addition of NBS over 60 min (71% by qNMR, 98:2 er). The product was isolated after column chromatography (100% CH_2_Cl_2_) to give a pale-orange oil (105 mg, 65% isolated yield). When performed at a 3 mmol scale (4× GP2) with an initial amount of 200 μL of water and an addition of NBS over 60 min, the yield by qNMR was 72%. The product was isolated after column chromatography (100% CH_2_Cl_2_) to give a pale-orange oil (374 mg, 58% isolated yield, 92:8 er). ^1^H NMR (400 MHz, CDCl_3_) δ 7.35–7.28 (m, 3H), 7.24–7.22 (m, 2H), 4.33 (tdd, *J* = 7.3, 6.2, 3.7 Hz, 1H), 3.83 (ddd, *J* = 12.4, 7.1, 3.7 Hz, 1H), 3.74 (dt, *J* = 12.4, 6.2 Hz, 1H), 3.27 (dd, *J* = 14.2, 7.3 Hz, 1H), 3.18 (dd, *J* = 14.2, 7.5 Hz, 1H), 2.01 (t, *J* = 6.9 Hz, 1H). ^13^C{^1^H} NMR (101 MHz, CDCl_3_) δ 137.8, 129.3, 128.8, 127.2, 66.2, 58.9, 41.5.

#### 2-Bromo-octan-1-ol^6^

2-Bromo-octan-1-ol was obtained following GP2 with an initial amount of 80 μL of water and an addition of NBS over 60 min (73% by qNMR, 95:5 er). The product was isolated after column chromatography (100% CHCl_3_) to give a colorless oil (92 mg, 59% isolated yield). ^1^H NMR (400 MHz, CDCl_3_) δ 4.18–4.12 (m, 1H), 3.82 (ddd, *J* = 11.8, 7.7, 3.9 Hz, 1H), 3.82 (dt, *J* = 12.3, 6.1 Hz, 1H), 1.99 (dd, *J* = 7.9, 5.7 Hz, 1H), 1.85 (q, *J* = 7.4 Hz, 2H), 1.47–1.39 (m, 1H), 1.38–1.25 (m, 7H), 0.89 (t, *J* = 6.7 Hz, 3H). ^13^C{^1^H} NMR (101 MHz, CDCl_3_) δ 67.5, 60.4, 35.0, 31.7, 28.8, 27.6, 22.7, 14.2.

#### 2-Bromo-dodecan-1-ol^16^

2-Bromo-dodecan-1-ol was obtained following GP2 with an initial amount of 80 μL of water and an addition of NBS over 60 min (44% by qNMR, 96:4 er). The product was isolated after column chromatography (100% CHCl_3_) to give a colorless oil (79 mg, 40% isolated yield). ^1^H NMR (400 MHz, CDCl_3_) δ 4.17–4.11 (m, 1H), 3.81 (dd, *J* = 12.3, 4.0 Hz, 1H), 3.74 (dd, *J* = 12.3, 6.9 Hz, 1H), 1.91 (bs, 1H), 1.87–1.81 (m, 2H), 1.58–1.49 (m, 1H), 1.45–1.38 (m, 1H), 1.33–1.23 (m, 14H), 0.87 (t, *J* = 6.8 Hz, 3H). ^13^C{^1^H} NMR (101 MHz, CDCl_3_) δ 67.4, 60.4, 35.0, 32.0, 29.71, 29.68, 29.54, 29.45, 29.1, 27.6, 22.8, 14.3.

#### 2-Bromo-pentan-1-ol^6^

2-Bromo-pentan-1-ol was obtained following GP2 with an initial amount of 100 μL of water and an addition of NBS over 75 min (65% by qNMR, 92:8 er). The product was isolated after column chromatography (100% CH_2_Cl_2_) to give a pale-yellow oil (59 mg, 47% isolated yield). ^1^H NMR (400 MHz, CDCl_3_) δ 4.16 (tdd, *J* = 7.1, 5.8, 4.0 Hz, 1H), 3.82 (dd, *J* = 12.3, 4.0 Hz, 1H), 3.74 (dd, *J* = 12.3, 7.0 Hz, 1H), 1.93 (bs, 1H), 1.86–1.78 (m, 2H), 1.65–1.52 (m, 1H), 1.52–1.39 (m, 1H), 0.94 (t, *J* = 7.4 Hz, 3H). ^13^C{^1^H} NMR (101 MHz, CDCl_3_) δ 67.5, 60.0, 37.0, 20.8, 13.6.

#### 2-Bromopropylbenzoate^17^

2-Bromopropylbenzoate was obtained following GP2 with an initial amount of 200 μL of water and an addition of NBS over 150 min (61% by qNMR, 76:24 er). This product was benzoylated following GP3 as the unprotected alcohol is volatile. The benzoylated product was isolated after column chromatography (100% CH_2_Cl_2_) and preparative TLC (95:5 hexane:EtOAc) to give a colorless oil (60 mg, 33% isolated yield). ^1^H NMR (400 MHz, CDCl_3_) δ 8.08–8.06 (m, 2H), 7.60–7.57 (m, 1H), 7.48–7.44 (m, 2H), 4.55–4.45 (m, 2H), 4.40–4.32 (m, 1H), 1.79 (d, *J* = 6.8 Hz, 3H). ^13^C{^1^H} NMR (101 MHz, CDCl_3_) δ 166.4, 133.7, 130.6, 130.2, 128.9, 69.7, 45.2, 23.0.

#### 2-Bromo-3-methylbutan-1-ol^7^

2-Bromo-3-methylbutan-1-ol was obtained following GP2 with an initial amount of 85 μL of water and an addition of NBS over 285 min (69% by qNMR, 95:5 er). The product was isolated after column chromatography (100% CH_2_Cl_2_) to give a pale-yellow oil (69 mg, 51% isolated yield). ^1^H NMR (400 MHz, CDCl_3_) δ 4.10 (ddd, *J* = 6.3, 5.6, 4.8 Hz, 1H), 3.84–3.79 (m, 2H), 2.02 (heptd, *J* = 6.7, 4.8 Hz, 1H), 1.95 (bs, 1H), 1.05 (d, *J* = 6.7 Hz, 3H), 1.02 (d, *J* = 6.6 Hz, 3H). ^13^C{^1^H} NMR (101 MHz, CDCl_3_) δ 68.4, 65.9, 31.6, 21.0, 19.2.

#### 2-Bromo-3-cyclohexylpropan-1-ol^6^

2-Bromo-3-cyclohexylpropan-1-ol was obtained following GP2 with an initial amount of 50 μL of water and an addition of NBS over 90 min at room temperature (72% by qNMR, 90:10 er). The product was isolated after column chromatography (100% CH_2_Cl_2_) to give a pale-yellow oil (102 mg, 62% isolated yield). ^1^H NMR (400 MHz, CDCl_3_) δ 4.25 (dddd, *J* = 10.4, 7.0, 4.5, 3.7 Hz, 1H), 3.81 (ddd, *J* = 12.2, 8.0, 3.7 Hz, 1H), 3.72 (ddd, *J* = 12.2, 7.0, 5.6 Hz, 1H), 2.03 (dd, *J* = 8.0, 5.6 Hz, 1H), 1.82–1.52 (m, 8H), 1.31–1.22 (m, 2H), 1.18–1.10 (m, 1H), 1.02–0.94 (m, 1H), 0.88–0.78 (m, 1H). ^13^C{^1^H} NMR (101 MHz, CDCl_3_) δ 67.9, 58.3, 47.4, 35.6, 33.8, 32.2, 26.6, 26.3, 26.1.

## Supporting Information Available

Experimental procedures and spectral and chromatographic data (PDF)
