# Supplementary material for: Organocatalytic Enantioselective α-Bromination of Aldehydes with N-Bromosuccinimide
Source: J Org Chem. 2022 May 26;87(12):7968–74. doi: 10.1021/acs.joc.2c00600 (PMC9207931; doi:10.1021/acs.joc.2c00600)
Supplement: Supplementary file 1 — jo2c00600_si_001.pdf [file jo2c00600_si_001.pdf]

Supporting Information

Organocatalytic Enantioselective α-Bromination of Aldehydes with *N*-Bromosuccinimide

George Hutchinson,* Carla Alamillo-Ferrer, Martín Fernández-Pascual, and Jordi Burés*

The University of Manchester, Department of Chemistry
Oxford Road, Manchester, M13 9PL, UK
E-mail: george.hutchinson@manchester.ac.uk, jordi.bures@manchester.ac.uk

Table of Contents

1. General Information S-2
2. General Procedures S-3
3. Procedures and Characterization Data S-4
4. Additional Experiments S-12
    4.1. Catalyst Brominations S-12
    4.2. Product Racemization S-15
    4.3. Reactions with an Instantaneous Injection of NBS S-17
    4.4. Uncatalyzed Reaction S-20
    4.5. Bromination of Propanal S-21
    4.6. Enantiomeric Ratio over the Slow Addition S-22
5. Optimization tables S-23
6. NMR spectra of brominated products S-25
7. Determination of the enantiomeric ratio of bromohydrin products S-32
8. References S-40

1. General Information

Commercially available aldehydes were carefully distilled under vacuum into an LN_2 trap immediately prior to use. The 3-cyclohexylpropanal was synthesized by Dess-Martin periodinane (DMP) oxidation of 3-cyclohexylpropanol following **GP1**. The *N*-bromosuccinimide (NBS) was recrystallized from water. The (*S*)-α,α-bis[3,5-bis(trifluoromethyl)-phenyl]-2-pyrrolidinemethanol trimethylsilyl ether (**3c**) and (*S*)-α,α-bis[3,5-bis(trifluoromethyl)-phenyl]-2-pyrrolidinemethanol *tert*-butyldimethylsilyl ether (**3e**) catalysts were purified from commercial sources by flash column chromatography (CH_2Cl_2) to remove any deprotected alcohol. All other reagents and solvents were used as purchased from Merck, Fluorochem, Alfa Aesar and TCI.

All NMR spectra were recorded on a Bruker AVII 500 MHz spectrometer or a Bruker AVIII HD 400 MHz spectrometer with BBO prodigy probe. ^{1}H NMR and ^{13}C NMR chemicals shifts (δ) are quoted in ppm relative to residual solvent peaks (for ^{1}H and ^{13}C respectively, given in ppm, for $CDCl_3$: 7.26, 77.16). Any non-deuterated NMR spectra were recorded after shimming on the solvent peak closest to the middle of the spectrum and are reported with respect to the shift of this solvent peak aligned with its position in $CDCl_3$ (for ^{1}H and ^{13}C respectively, given in ppm, for HFIP: 4.49, 69.20). Chiral HPLC was carried out on an Agilent 1260 Infinity II LC equipped with a diode array detector. The column, solvent system and flow rate are noted with the chromatograms. Slow additions were carried out using a Harvard Apparatus standard infuse/withdraw pump 11 elite programmable syringe pump calibrated to the syringe, a Henke-Sans-Wolfe Air-tight 2.5 mL. The brominating agent was added as a stock solution through PTFE tubing with an internal diameter of 0.50 mm. All bromination reactions were carried out in a STEM Integrity 10 set to the desired temperature.

Flash column chromatography was performed using 230-400 mesh silica, with the indicated solvent system according to standard techniques. Analytical thin-layer chromatography (TLC) and preparative thin-layer chromatography were performed on precoated glass-backed silica gel plates (Supelco TLC Silica gel 60 F_{254}). Visualization of the developed chromatogram was performed by UV absorbance (254 nm) or anisaldehyde stain.

The time of addition and amount of water required for each substrate were optimized following the procedure described for chlorination in our previous work.[1] Yields were calculated after reduction of the α-bromoaldehydes to the corresponding bromohydrins using qNMR with an internal standard (either 1,1,2,2-tetrachloroethane - TCE, 1,3-dinitrobenzene - DNB, 1,3,5-trimethoxybenzene – TMB or 4-nitrotrifluromethylbenzene - NTB). Experiments were repeated and the products purified to determine an isolated yield.

2. General Procedures

General Procedure 1 (GP1): DMP oxidation of 3-cyclohexylpropan-1-ol[2]

To a stirred solution of the alcohol (2.0 g, 14 mmol, 1 equiv) in dry CH_2Cl_2 (50 mL, 0.28 M) under N_2 was added DMP (7.2 g, 17 mmol, 1.2 equiv). The reaction mixture was stirred for 3 h before Et_2O (200 mL) and sat. $NaHCO_{3(aq)}$ (100 mL) were added. After stirring for 10 min, the mixture was filtered through a short plug of celite and transferred to a separating funnel. The organic phase was washed with sat. $NaHCO_{3(aq)}$ (2 x 100 mL) and brine (50 mL) before the organic phase was collected, dried over $MgSO_4$, and concentrated under reduced pressure. The crude residue was purified by flash column chromatography (9:1 Hexane:EtOAc) to afford a colorless oil (1.70 g, 12 mmol, 86%).

General Procedure 2 (GP2): Bromination of aldehydes

Solutions of aldehyde (1.88 mmol in 500 µL, 2.5 equiv), (S)-α,α-bis[3,5-bis(trifluoromethyl)-phenyl]-2-pyrrolidinemethanol tert-butyldimethylsilyl ether **3e** (0.015 mmol in 500 µL, 2 mol%) and H_2O (determined amount in 500 µL) in HFIP were added to a stirred vial containing HFIP (500 µL) at 4 °C. The reaction mixture was stirred for 2 min before a solution of NBS (0.75 mmol in 1000 µL, 1 equiv) in HFIP was added over the determined time. Immediately after the end of the addition, the reaction mixture was transferred to a stirred flask containing MeOH (1 mL) and CH_2Cl_2 (1 mL) before $NaBH_4$ (approx. 5 equiv) was added. This mixture was stirred for 2 min before sat. $NH_4Cl_{(aq)}$ (5 mL), H_2O (5 mL) and a stock solution of internal standard were added. The mixture was extracted with CH_2Cl_2 (4 x 15 mL), before the combined organic phase was washed with brine (15 mL), dried over $MgSO_4$, and concentrated under reduced pressure (care should be taken as some of the bromohydrins, particularly 2-bromo-3-methylbutan-1-ol, 2-bromo-pentan-1-ol and 2-bromo-propan-1-ol, are volatile). The products were isolated after purification by flash column chromatography.

General Procedure 3 (GP3): Benzoylation of alcohols

The crude or purified product from **GP2** was dissolved in dry CH_2Cl_2 (10 mL, 0.075 M) before BzCl (3 mmol, 347 µL, 4 equiv) was added. NEt_3 (3 mmol, 413 µL, 4 equiv) was added dropwise over 30 s. The reaction mixture was stirred overnight before sat. $NaHCO_{3(aq)}$ (10 mL) was added. The mixture was transferred to a separating funnel and the organic phase was collected, dried over $MgSO_4$, and concentrated under reduced pressure. The crude reside was purified by flash column chromatography or preparative TLC.

General Procedure 4 (GP4): Synthesis of racemic bromohydrins

To a stirred solution of the aldehyde (3.75 mmol, 1 equiv) in CH_2Cl_2 (10 mL, 0.375 M) was added NBS (800 mg, 4.5 mmol, 1.2 equiv) and DL-proline (86 mg, 0.75 mmol, 20 mol%) at room temperature. The reaction mixture was stirred for 2 h before being transferred to a stirred vial containing MeOH (5 mL) and $NaBH_4$ (approx. 5 equiv). The reduction was stirred for 2 min before sat. $NH_4Cl_{(aq)}$ (10 mL) and H_2O (5 mL) were added. The mixture was extracted with CH_2Cl_2 (4 x 30 mL), before the combined organic phase was washed with brine (30 mL), dried over $MgSO_4$, and concentrated under reduced pressure.

3. Procedures and Characterization Data

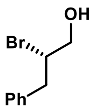

2-Bromo-3-phenylpropan-1-ol

The title compound was obtained following **GP2** with an initial amount of 50 µL of water and an addition of NBS over 60 min (71%, 98:2 er). The spectroscopic data matches that from the literature.[3] Quantitative NMR with an internal standard (TCE, 0.25 mmol) indicated that there was 0.53 mmol of monobrominated alcohol (Figure S1). The product was isolated after column chromatography (100% CH_2Cl_2) to give a pale orange oil (105 mg, 65%). **^{1}H NMR (400 MHz, $CDCl_3$) δ** 7.35 – 7.28 (m, 3H), 7.24 – 7.22 (m, 2H), 4.33 (tdd, *J* = 7.3, 6.2, 3.7 Hz, 1H), 3.83 (ddd, *J* = 12.4, 7.1, 3.7 Hz, 1H), 3.74 (dt, *J* = 12.4, 6.2 Hz, 1H), 3.27 (dd, *J* = 14.2, 7.3 Hz, 1H), 3.18 (dd, *J* = 14.2, 7.5 Hz, 1H), 2.01 (t, *J* = 6.9 Hz, 1H). **^{13}C{^{1}H} NMR (101 MHz, $CDCl_3$) δ** 137.8, 129.3, 128.8, 127.2, 66.2, 58.9, 41.5.

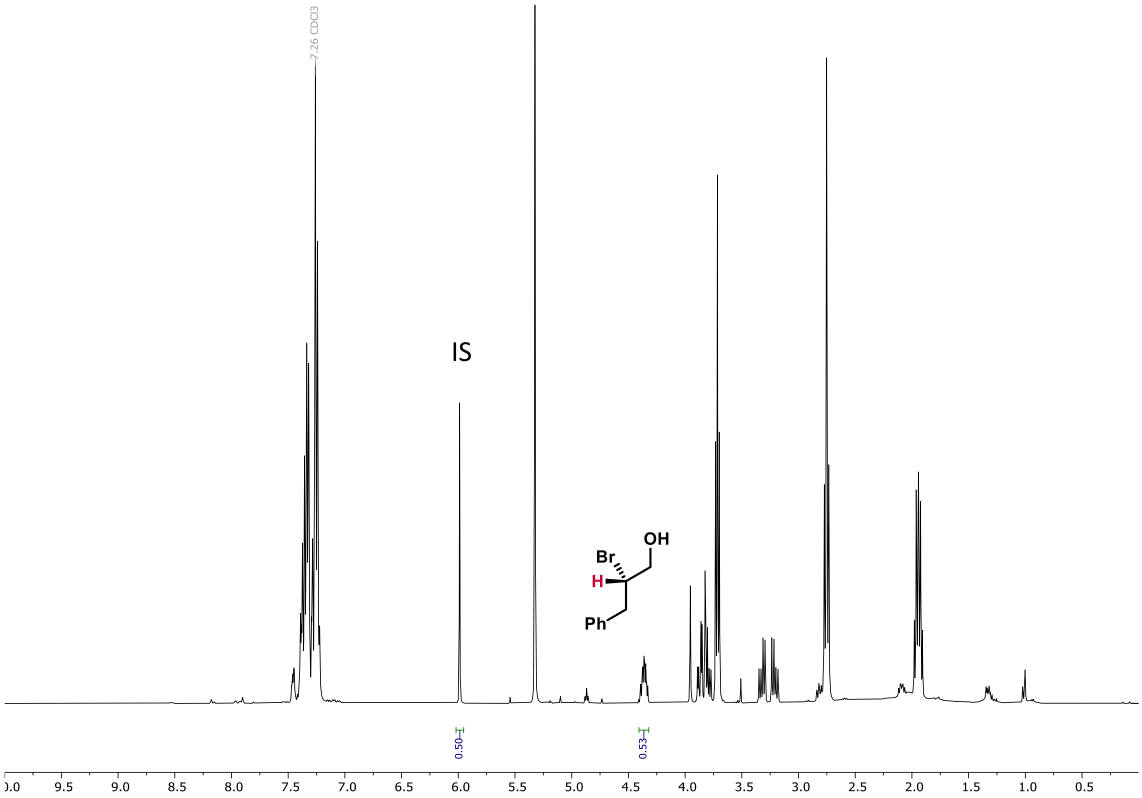

Figure S1. Quantitative NMR of the crude reaction mixture after $NaBH_4$ reduction showing the yield of 2-bromo-3-phenylpropan-1-ol

Large scale bromination of hydrocinnamaldehyde

Solutions of hydrocinnamaldehyde (7.52 mmol in 2.0 mL, 2.5 equiv), (*S*)-α,α-bis[3,5-bis(trifluoromethyl)-phenyl]-2-pyrrolidinemethanol *tert*-butyldimethylsilyl ether **3e** (0.06 mmol in 2.0 mL, 2 mol%) and H_2O (200 µL in 2.0 mL) in HFIP were added to a stirred vial containing HFIP (2.0 mL) at 4 °C. The reaction mixture was stirred for 2 min before a solution of NBS (3.0 mmol in 4.0 mL, 1.0 equiv) in HFIP was added over 1 h. Immediately after the end of the addition, the reaction mixture was transferred to a stirred flask containing MeOH (4 mL) and CH_2Cl_2 (4 mL) before $NaBH_4$ (approx. 5 equiv) was added. This mixture was stirred for 2 min before sat. $NH_4Cl_{(aq)}$ (20 mL), H_2O (20 mL) and a stock solution of internal standard were added (1.0 mmol TMB in 1 mL $CHCl_3$). The mixture was extracted with CH_2Cl_2 (4 x 60 mL), before the combined organic phase was washed with brine (60 mL), dried over $MgSO_4$, and concentrated under reduced pressure. Quantitative ^{1}H NMR indicated that there was 2.15 mmol of monobrominated alcohol, a yield of 72%. The product was isolated after purification by flash column chromatography (100% CH_2Cl_2) to give a pale-orange oil (374 mg, 58%).

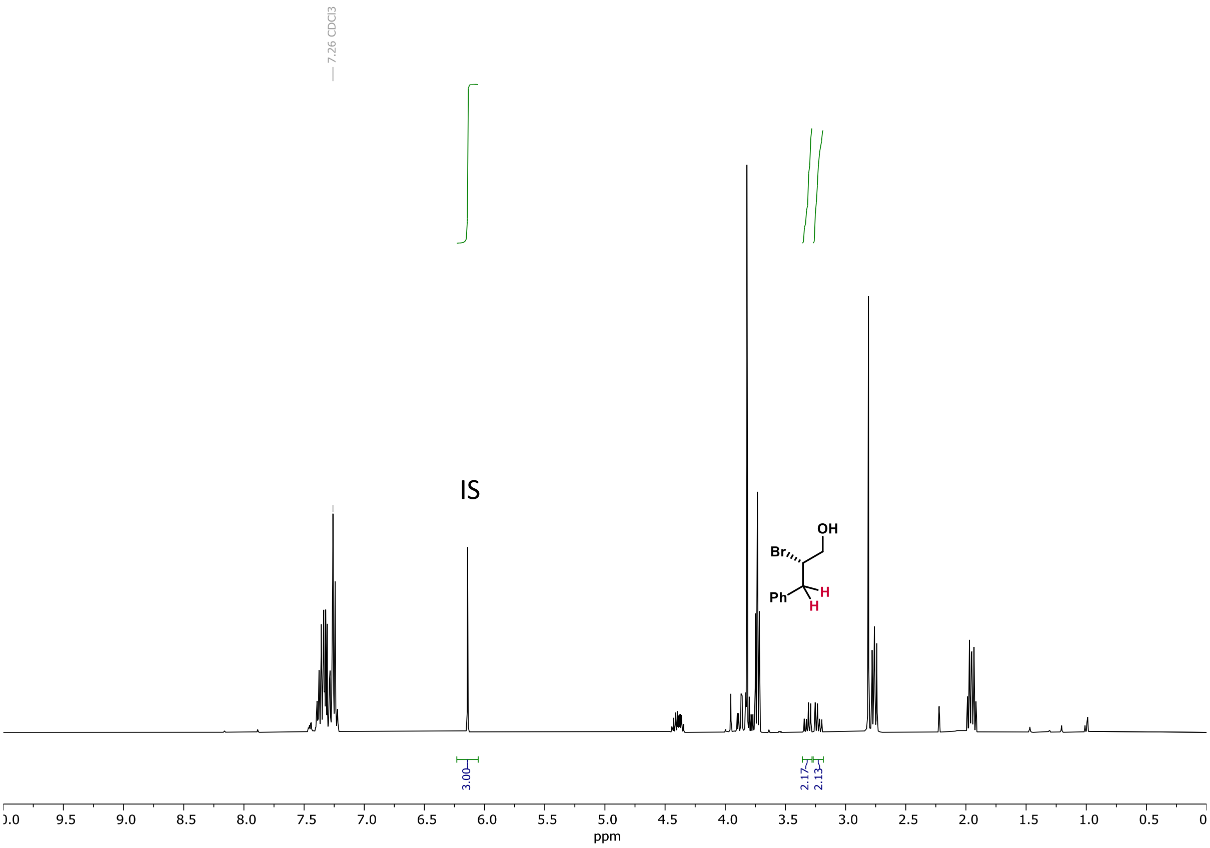

Figure S2. Quantitative NMR of the large-scale crude reaction mixture after $NaBH_4$ reduction showing the yield of 2-bromo-3-phenylpropan-1-ol

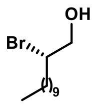

2-Bromo-dodecan-1-ol

The title compound was obtained following **GP2** with an initial amount of 80 µL of water and an addition of NBS over 60 min (44%, 96:4 er). The spectroscopic data matches that from the literature.[4] Quantitative NMR with an internal standard (TMB, 0.25 mmol) indicated that there was 0.33 mmol of monobrominated alcohol (Figure S3). The product was isolated after column chromatography (100% $CHCl_3$) to give a colorless oil (79 mg, 40%). **^{1}H NMR (400 MHz, $CDCl_3$) δ** 4.17 – 4.11 (m, 1H), 3.81 (dd, *J* = 12.3, 4.0 Hz, 1H), 3.74 (dd, *J* = 12.3, 6.9 Hz, 1H), 1.91 (bs, 1H), 1.87 – 1.81 (m, 2H), 1.58 – 1.49 (m, 1H), 1.45 – 1.38 (m, 1H), 1.33 – 1.23 (m, 14H), 0.87 (t, *J* = 6.8 Hz, 3H). **^{13}C{^{1}H} NMR (101 MHz, $CDCl_3$) δ** 67.4, 60.4, 35.0, 32.0, 29.71, 29.68, 29.54, 29.45, 29.1, 27.6, 22.8, 14.3.

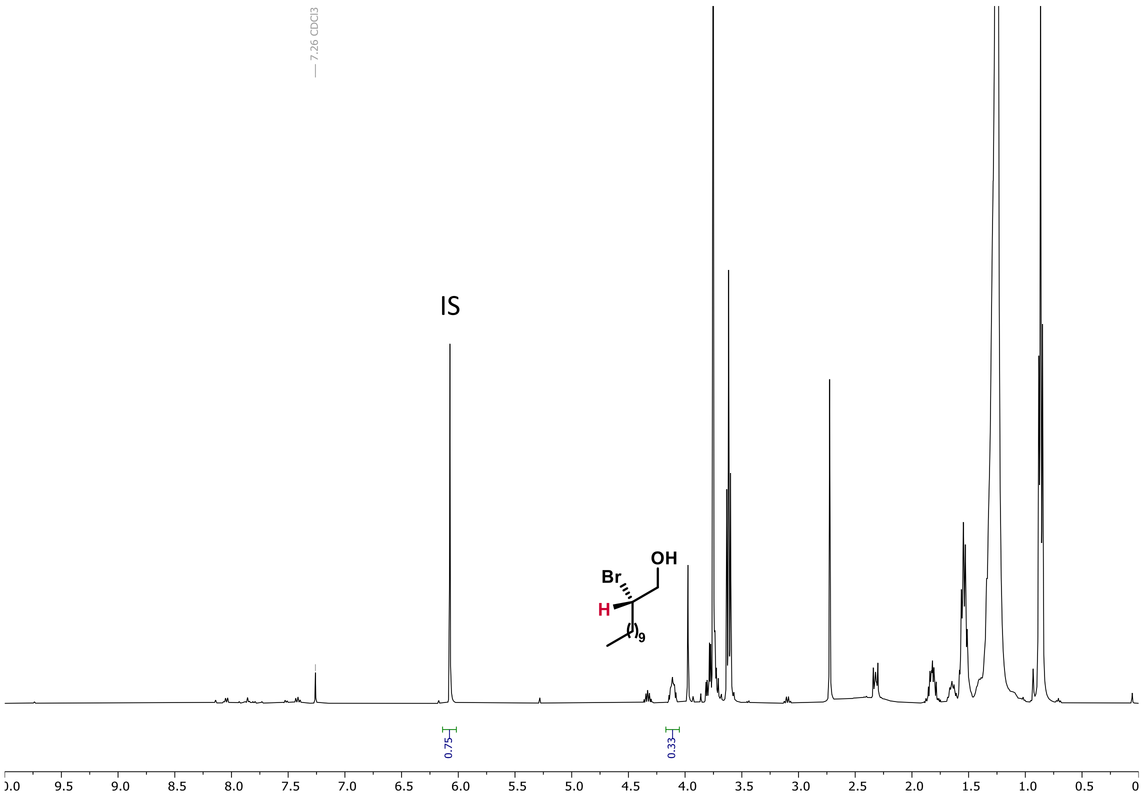

Figure S3. Quantitative NMR of the crude reaction mixture after $NaBH_4$ reduction showing the yield of 2-bromo-dodecan-1-ol

2-Bromo-octan-1-ol

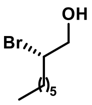

The title compound was obtained following **GP2** with an initial amount of 80 µL of water and an addition of NBS over 60 min (73%, 95:5 er). The spectroscopic data matches that from the literature.[5] Quantitative NMR with an internal standard (TCE, 0.375 mmol) indicated that there was 0.55 mmol of monobrominated alcohol (Figure S4). The product was isolated after column chromatography (100% $CHCl_3$) to give a colorless oil (92 mg, 59%). **^{1}H NMR (400 MHz, $CDCl_3$) δ** 4.18 – 4.12 (m, 1H), 3.82 (ddd, *J* = 11.8, 7.7, 3.9 Hz, 1H), 3.82 (dt, *J* = 12.3, 6.1 Hz, 1H), 1.99 (dd, *J* = 7.9, 5.7 Hz, 1H), 1.85 (q, *J* = 7.4 Hz, 2H), 1.47 – 1.39 (m, 1H), 1.38 – 1.25 (m, 7H), 0.89 (t, *J* = 6.7 Hz, 3H). **^{13}C{^{1}H} NMR (101 MHz, $CDCl_3$) δ** 67.5, 60.4, 35.0, 31.7, 28.8, 27.6, 22.7, 14.2.

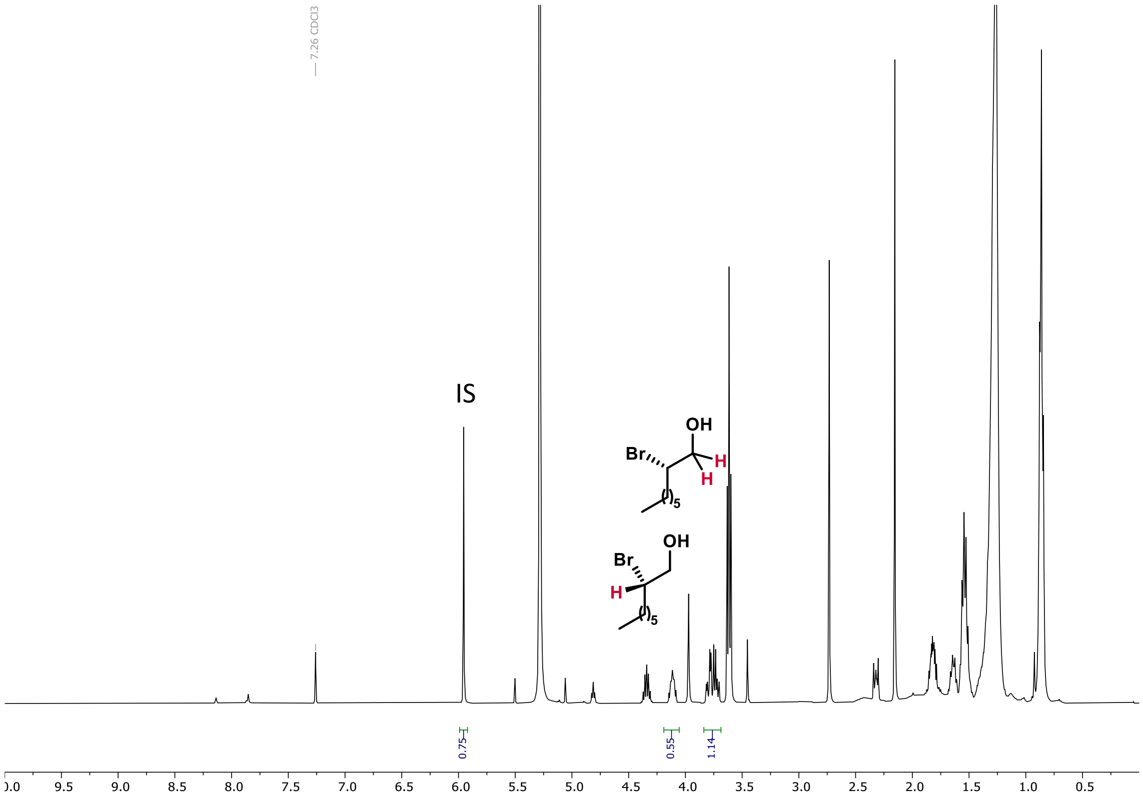

Figure S4. Quantitative NMR of the crude reaction mixture after $NaBH_4$ reduction showing the yield of 2-bromo-octan-1-ol

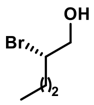

2-Bromo-pentan-1-ol

The title compound was obtained following **GP2** with an initial amount of 100 µL of water and an addition of NBS over 75 min (65%, 92:8 er). The spectroscopic data matches that from the literature.[5] Quantitative NMR with an internal standard (TMB, 0.25 mmol) indicated that there was 0.49 mmol of monobrominated alcohol (Figure S5). The product was isolated after column chromatography (100% CH_2Cl_2) to give a pale-yellow oil (59 mg, 47%). **^{1}H NMR (400 MHz, $CDCl_3$) δ** 4.16 (tdd, *J* = 7.1, 5.8, 4.0 Hz, 1H), 3.82 (dd, *J* = 12.3, 4.0 Hz, 1H), 3.74 (dd, *J* = 12.3, 7.0 Hz, 1H), 1.93 (bs, 1H), 1.86 – 1.78 (m, 2H), 1.65 – 1.52 (m, 1H), 1.52 – 1.39 (m, 1H), 0.94 (t, *J* = 7.4 Hz, 3H). **^{13}C{^{1}H} NMR (101 MHz, $CDCl_3$) δ** 67.5, 60.0, 37.0, 20.8, 13.6.

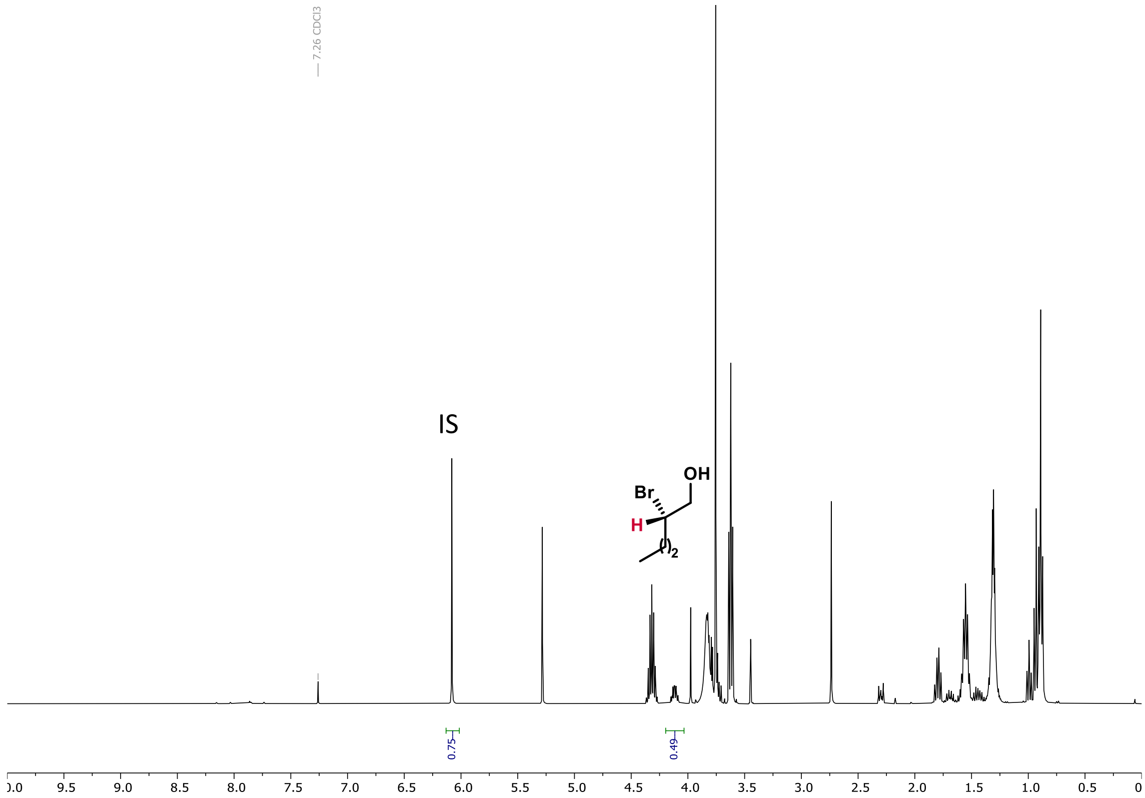

Figure S5. Quantitative NMR of the crude reaction mixture after $NaBH_4$ reduction showing the yield of 2-bromo-pentan-1-ol

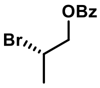

2-Bromopropylbenzoate

The title compound was obtained following **GP2** with an initial amount of 200 µL of water and an addition of NBS over 150 min (61%, 76:24 er). The qNMR to determine the yield was taken directly from the reduction mixture in methanol. The peaks in the crude ^{1}H NMR spectra match those from the literature.[6] Quantitative NMR with an internal standard (DNB, 0.60 mmol) indicated that there was 0.46 mmol of monobrominated alcohol (Figure S6). This product was isolated after benzoylation following **GP3** as the unprotected alcohol is volatile. The benzoylated product was isolated after column chromatography (100% CH_2Cl_2) and preparative TLC (95:5 Hexane:EtOAc) to give a colorless oil (60 mg, 33%). **^{1}H NMR (400 MHz, $CDCl_3$) δ** 8.08 – 8.06 (m, 2H), 7.60 – 7.57 (m, 1H), 7.48 – 7.44 (m, 2H), 4.55 – 4.45 (m, 2H), 4.40 – 4.32 (m, 1H), 1.79 (d, *J* = 6.8 Hz, 3H). **^{13}C{^{1}H} NMR (101 MHz, $CDCl_3$) δ** 166.4, 133.7, 130.6, 130.2, 128.9, 69.7, 45.2, 23.0.

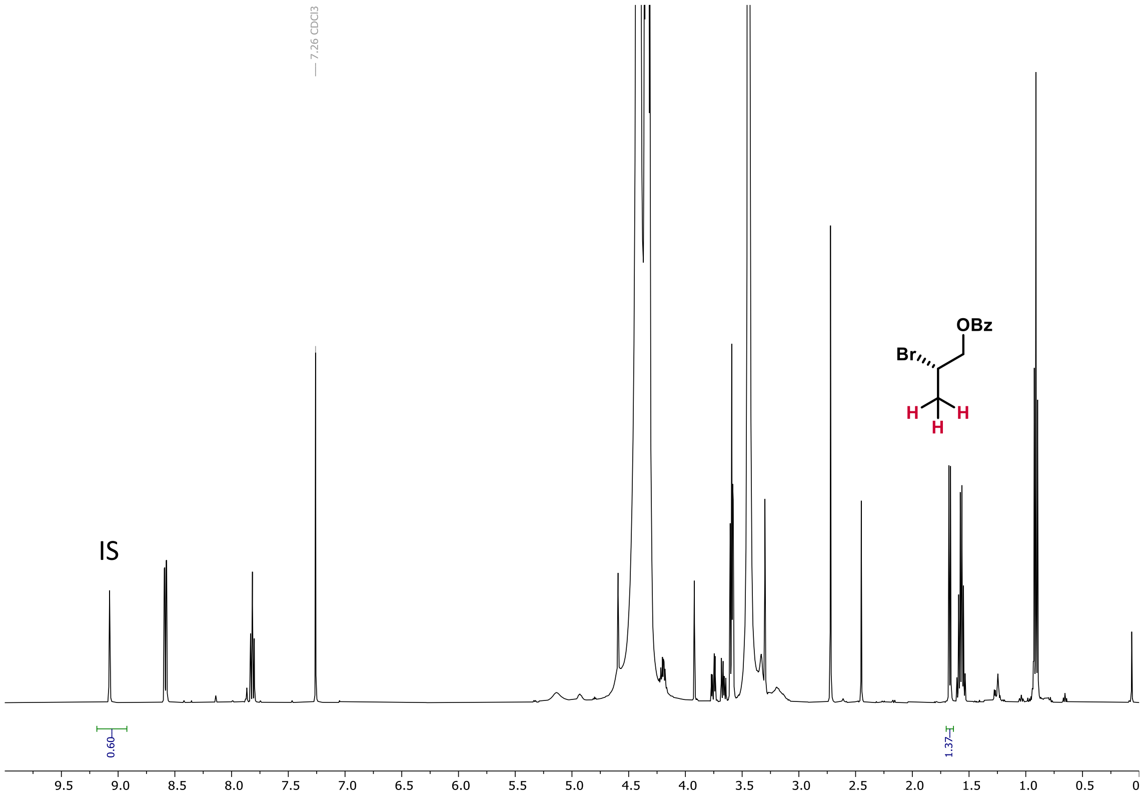

Figure S6. Quantitative NMR of the crude reaction mixture after $NaBH_4$ reduction showing the yield of 2-bromo-propan-1-ol

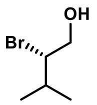

2-Bromo-3-methylbutan-1-ol

The title compound was obtained following **GP2** with an initial amount of 85 µL of water and an addition of NBS over 285 min (69%, 95:5 er). The spectroscopic data matches that from the literature.[3] Quantitative NMR with an internal standard (TCE, 0.375 mmol) indicated that there was 0.52 mmol of monobrominated alcohol (Figure S7). The product was isolated after column chromatography (100% CH_2Cl_2) to give a pale-yellow oil (69 mg, 51%). **^{1}H NMR (400 MHz, $CDCl_3$) δ** 4.10 (ddd, *J* = 6.3, 5.6, 4.8 Hz, 1H), 3.84 – 3.79 (m, 2H), 2.02 (heptd, *J* = 6.7, 4.8 Hz, 1H), 1.95 (bs, 1H), 1.05 (d, *J* = 6.7 Hz, 3H), 1.02 (d, *J* = 6.6 Hz, 3H). **^{13}C{^{1}H} NMR (101 MHz, $CDCl_3$) δ** 68.4, 65.9, 31.6, 21.0, 19.2.

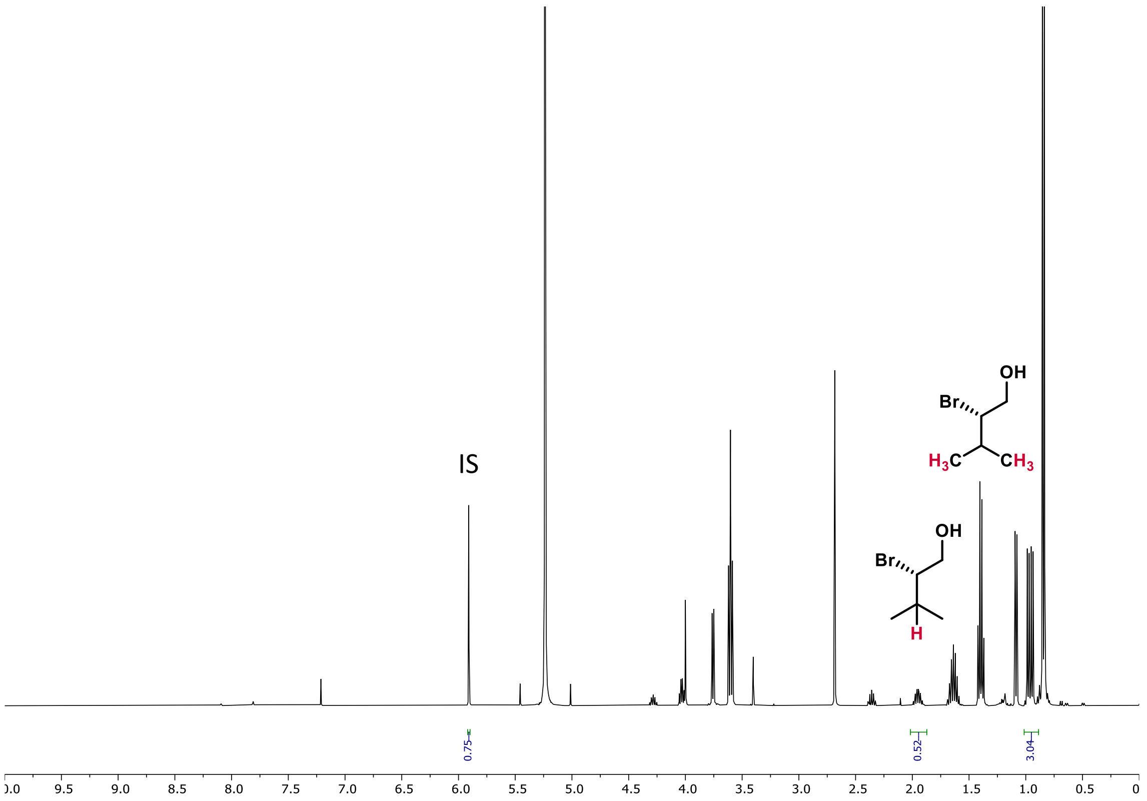

Figure S7. Quantitative NMR of the crude reaction mixture after $NaBH_4$ reduction showing the yield of 2-bromo-3-methylbutan-1-ol

2-Bromo-3-cyclohexylpropan-1-ol

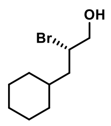

The title compound was obtained following **GP2** with an initial amount of 50 µL of water and an addition of NBS over 90 min at room temperature (72%, 90:10 er). The spectroscopic data matches that from the literature.[5] Quantitative NMR with an internal standard (TMB, 0.25 mmol) indicated that there was 0.54 mmol of monobrominated alcohol (Figure S8). The product was isolated after column chromatography (100% CH_2Cl_2) to give a pale-yellow oil (102 mg, 62%). **^{1}H NMR (400 MHz, $CDCl_3$) δ** 4.25 (dddd, *J* = 10.4, 7.0, 4.5, 3.7 Hz, 1H), 3.81 (ddd, *J* = 12.2, 8.0, 3.7 Hz, 1H), 3.72 (ddd, *J* = 12.2, 7.0, 5.6 Hz, 1H), 2.03 (dd, *J* = 8.0, 5.6 Hz, 1H), 1.82 – 1.52 (m, 8H), 1.31 – 1.22 (m, 2H), 1.18 – 1.10 (m, 1H), 1.02 – 0.94 (m, 1H), 0.88 – 0.78 (m, 1H). **^{13}C{^{1}H} NMR (101 MHz, $CDCl_3$) δ** 67.9, 58.3, 47.4, 35.6, 33.8, 32.2, 26.6, 26.3, 26.1.

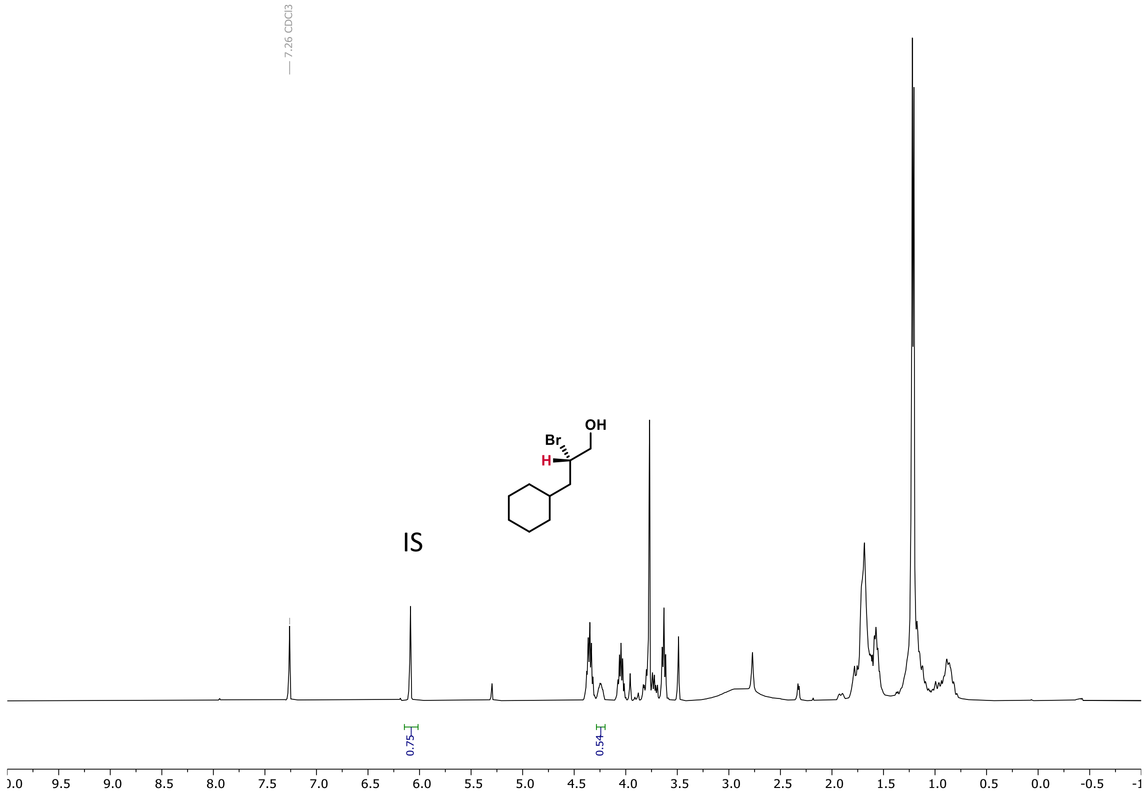

Figure S8. Quantitative NMR of the crude reaction mixture after $NaBH_4$ reduction showing the yield of 2-bromo-3-cyclohexyllpropan-1-ol

4. Additional Experiments

4.1 Catalyst Brominations

Stability of catalyst 3b when mixed with NBS

We explored the stability of the Jørgensen-Hayashi catalyst **3b** in HFIP when mixed with 2.0 equivalents of NBS. We mixed (*S*)-α,α-bis[3,5-bis(trifluoromethyl)-phenyl]-2-pyrrolidinemethanol trimethylsilyl ether (0.06 mmol, **3b**) with *N*-bromosuccinimide (0.12 mmol) in HFIP (0.6 mL). We took ^{1}H NMR spectra immediately after the addition of NBS, after 4h and after 16 h.

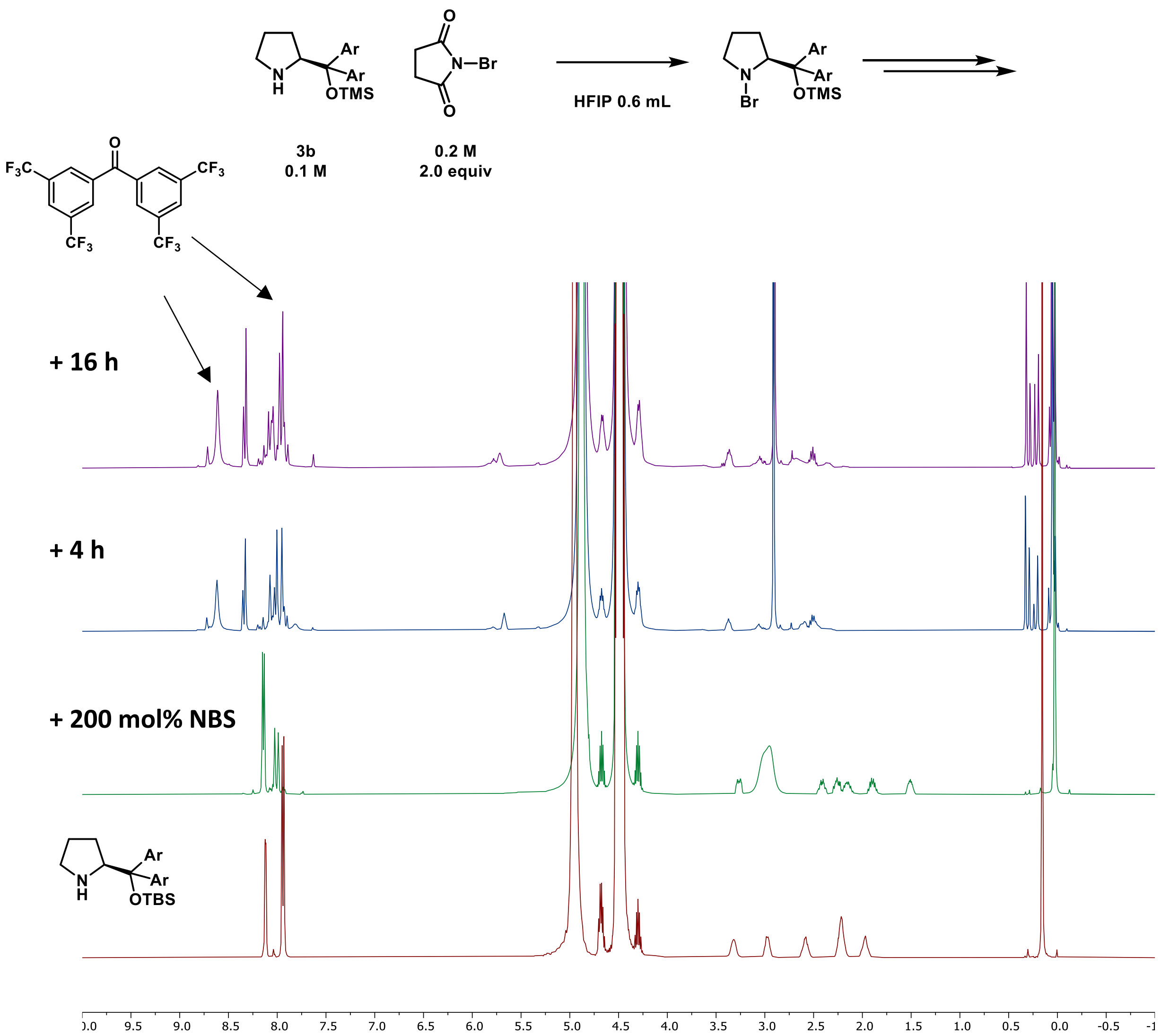

Figure S9. Stacked ^{1}H NMR spectra showing the decomposition of the brominated Ar,Ar-OTMS catalyst in HFIP

We found that this catalyst had suffered irreversible decomposition over less than four hours when exposed to an excess of NBS (Figure S9). We did not characterize the products of the decomposition apart from bis(3,5-bis(trifluoromethyl)phenyl)methanone. This species is indicative of a Grob-type fragmentation of the amine.[1]

Stability of catalyst 3e when mixed with NBS

We explored the stability of the Jørgensen-Hayashi catalyst **3e** in HFIP when mixed with 2.2 equivalents of NBS. We mixed (*S*)-α,α-bis[3,5-bis(trifluoromethyl)-phenyl]-2-pyrrolidinemethanol *tert*-butyldimethylsilyl ether (0.06 mmol, **3e**) with *N*-bromosuccinimide (0.132 mmol) in HFIP (0.6 mL). We monitored the reaction by collecting sequential ^{1}H NMR. We included TCE (0.05 M) as an internal standard.

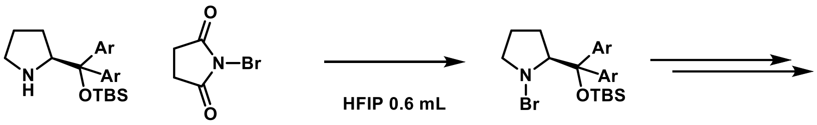

3b **0.1 M** — **0.22 M** **2.2 equiv**

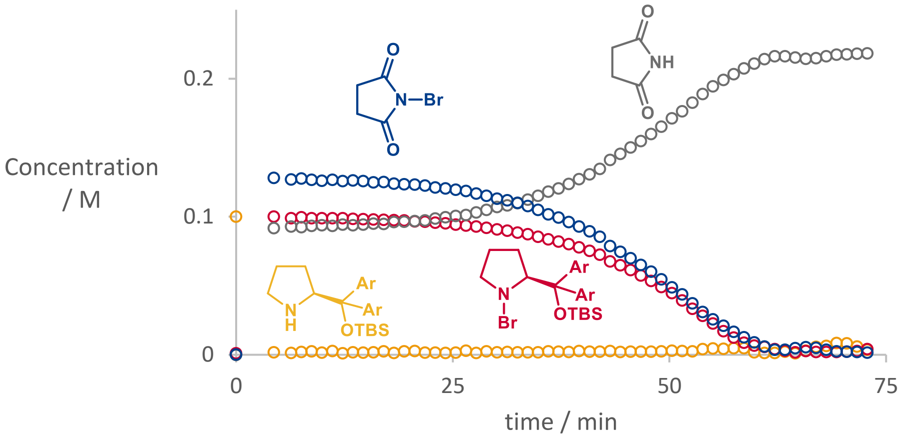

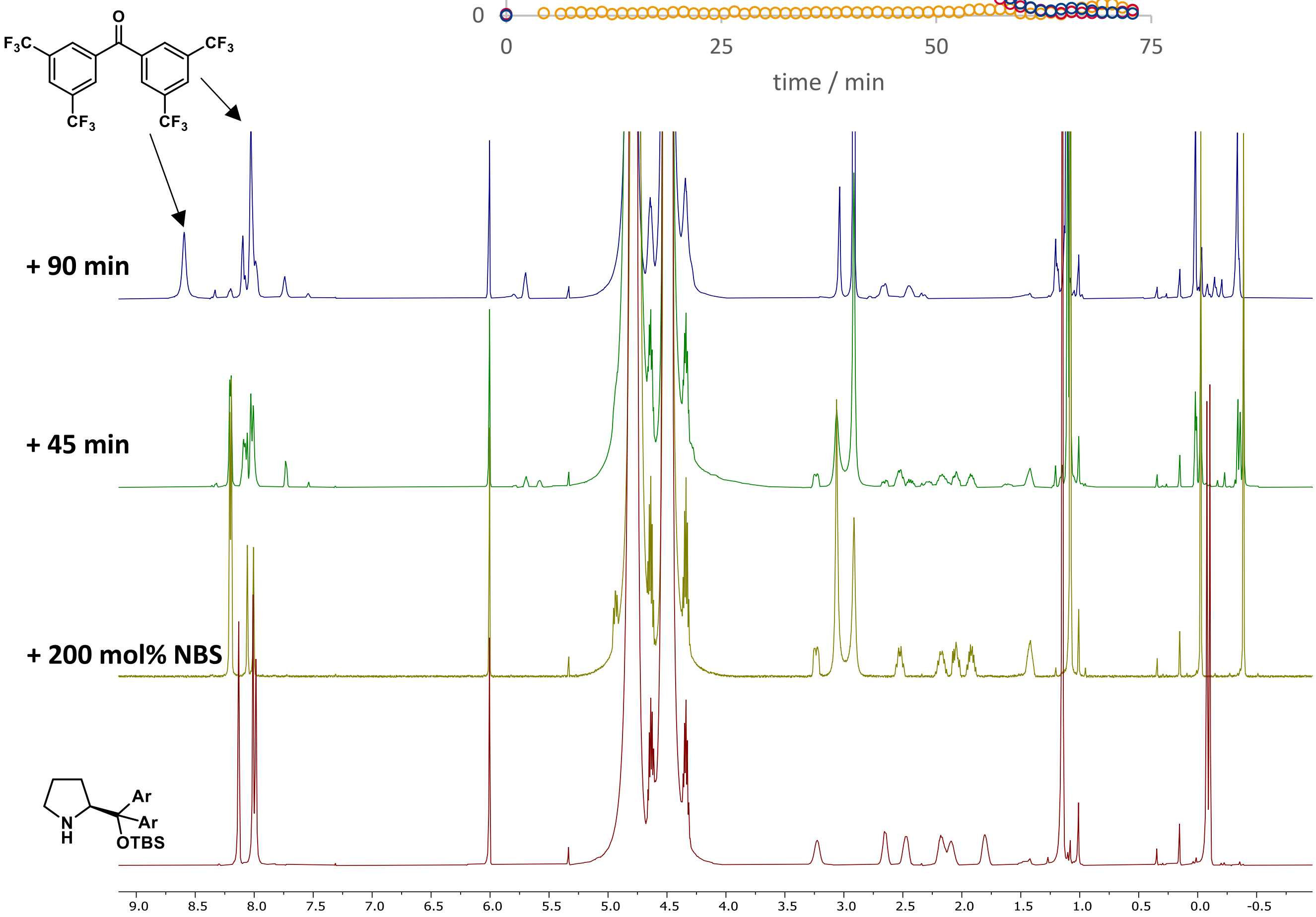

Figure S10. Brominated Ar,Ar-OTBS catalyst decomposes rapidly and NBS continues being consumed. The initial reaction between the catalyst and the NBS occurred faster than the first NMR spectra after the NBS addition could be recorded.

We found that that catalyst is completely decomposed after approximately 1 h, and that the decomposition gets faster over time (Figure S10). The excess NBS was consumed over the same time, suggesting that the products of the catalyst decomposition can also be brominated. As for catalyst **3b**, we observed aromatic signals at 8.58 and 8.04 ppm, visible in the spectra taken after 90 minutes, that are from bis(3,5-bis(trifluoromethyl)phenyl)methanone, indicative of a Grob-type fragmentation of the brominated catalyst. We did not characterize any of the other decomposition products.

4.2 Product Racemization

We tried to identify the cause of the comparatively poor enantiomeric excesses that we had observed in some of the bromination reactions. We carried out a reaction following **GP2** with catalyst **3b**, a 90 min addition of NBS and 100 µL of added water using hydrocinnamaldehyde as the substrate. After the end of the slow addition of the NBS, we allowed the reaction mixture to stir unquenched. We took three samples, immediately after the end of the addition, after half an hour and after 16 h. To determine if the enantiomeric excess of the product changed over time, we measured the enantiomeric excess of the product in each sample after reduction to the corresponding bromohydrin. We have indicated approximate conversions to the mono- and di-brominated aldehydes on the reaction scheme. These conversions are based on the ratio of the integrations of the alcohols of the starting material and the mono- and di-brominated products in the ^{1}H NMR spectra taken of the first sample after reduction.

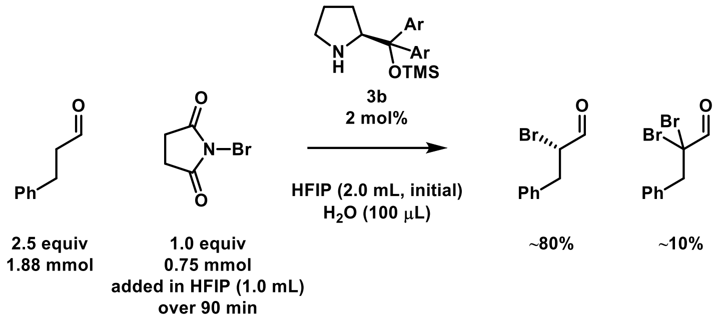

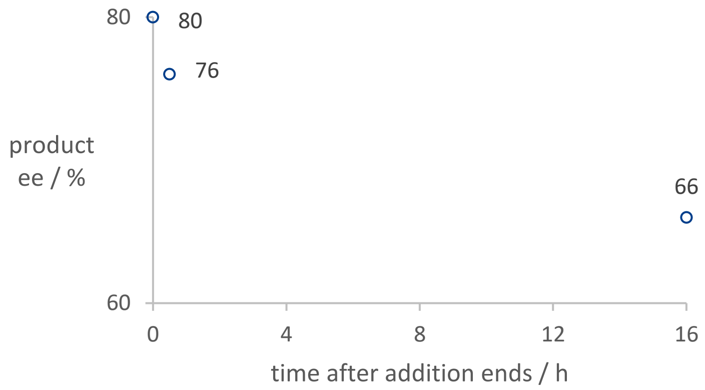

Figure S11. The product loses enantiomeric excess in the reaction media

The enantiomeric excess of the product decreases over time (Figure S11). We suspect that this relatively rapid reduction in ee, from 80 to 66% over 16 hours, could explain the relatively low enantiomeric excess we observed for some of the substrates. To identify the potential species that causes the product racemization, we repeated the reaction and isolated the monobrominated aldehyde (0.35 mmol per experiment) after the addition of NBS. After purification of the monobrominated aldehyde by column chromatography (100% CH_2Cl_2), the enantiomeric excess was 8% lower than the enantiomeric excess measured when the $NaBH_4$ reduction was carried out immediately at the end of the addition. In two different experiments, we mixed the isolated monobrominated aldehyde with catalyst **3b** (5 mol%) and with NHS (1.0 equiv) (Figure S12).

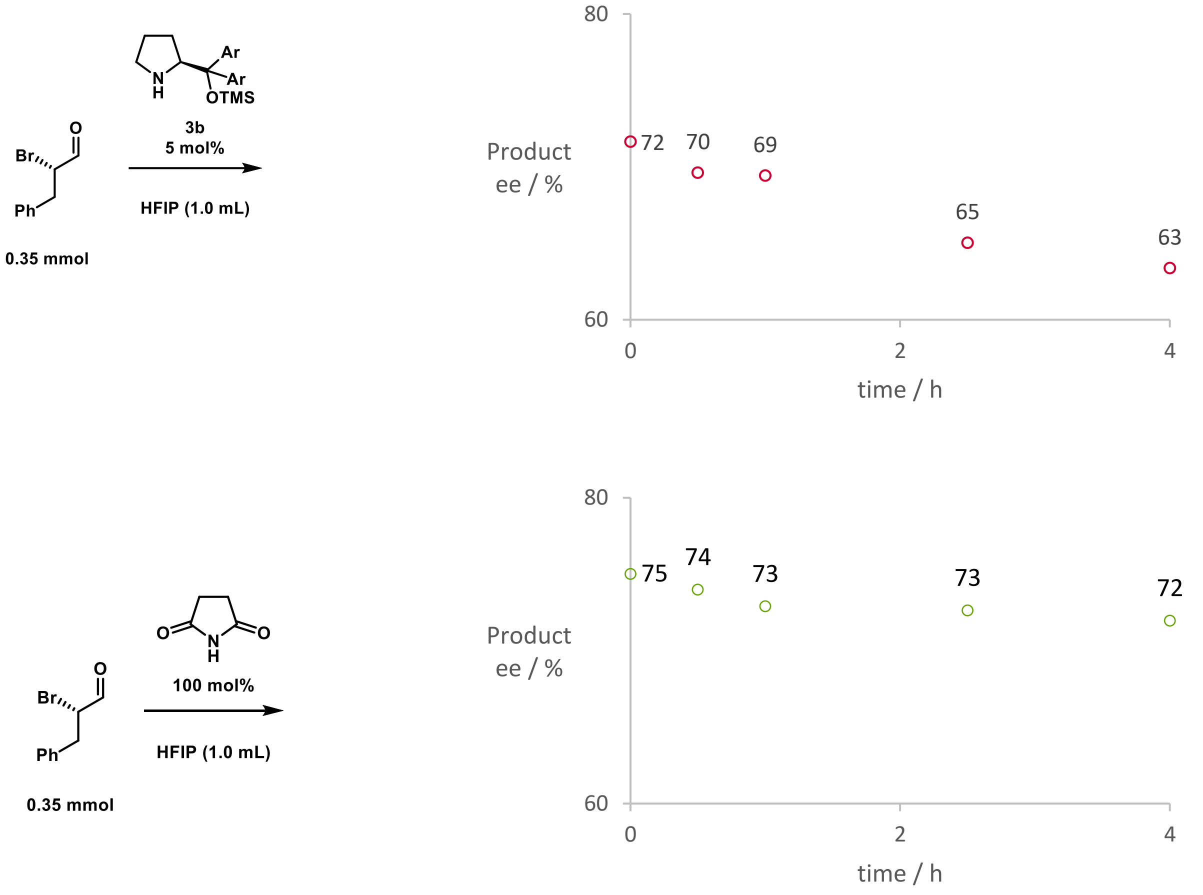

Figure S12. When isolated, the product loses enantiomeric excess when mixed with a secondary amine or succinimide

We observed a reduction of the enantiomeric excess of the product in both cases (Figure S12). The reduction in the enantiomeric excess was faster when the product was mixed with 5 mol% of catalyst **3b** than with 1 equiv NHS, though both experiments demonstrated significant losses in enantiomeric excess over 4 h.

4.3 Reactions with an instantaneous injection of NBS

We tested instantaneous injections of NBS in case the catalyst was sufficiently protected when condensed with the aldehyde to avoid bromination. We added a stock solution of catalyst **3b** (100 µL of a 0.03 M solution in HFIP) to a mixture of hydrocinnamaldehyde (0.38 mmol) and NBS (0.15 mmol) in HFIP (500 µL) to initiate the reaction. We monitored the reaction by taking sequential ^{1}H NMR spectra and compared the integrals of the aldehyde protons on the starting material (9.72 ppm), mono- (9.43 ppm) and dibrominated aldehydes (9.26 ppm). We observed that most of the catalyst (90%) was brominated in less than 5 minutes after the catalyst addition, but we also observed some of the iminium of hydrocinnamaldehyde and catalyst **3b** (10%) and the reaction proceeded. Around 30% of the NBS was consumed over 12 h.

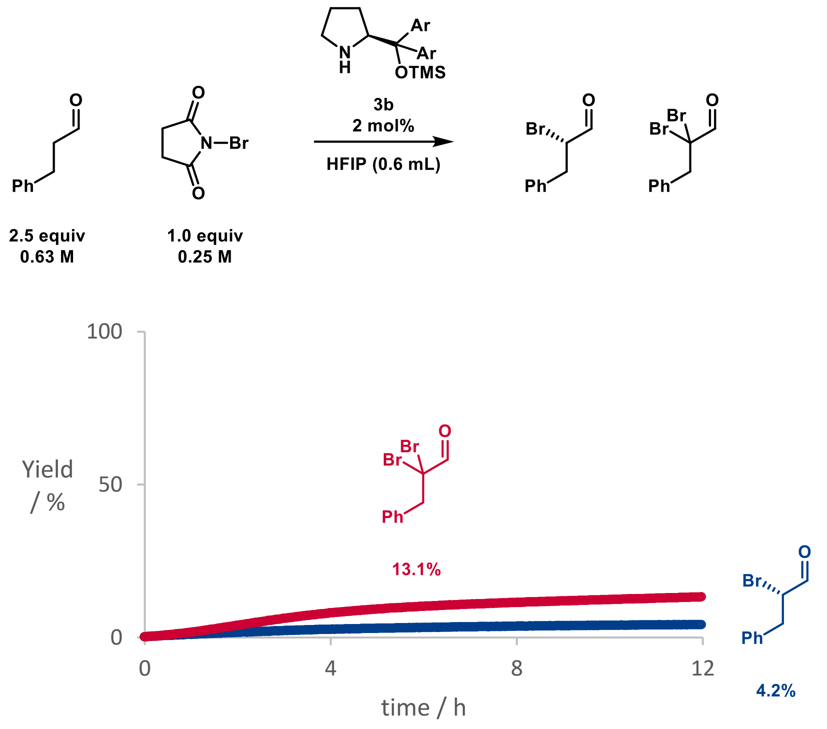

Figure S13. The yield of the reaction was very low after 12 h and most of the product was undesired dibrominated aldehyde.

The overall yield was quite poor (17.3%; 13.1% dibrominated and 4.2% monobrominated aldehyde, Figure S13). To improve this yield, we repeated the reaction with succinimide (0.15 mmol) added from the beginning of the reaction to shift the equilibrium between free catalyst and brominated catalyst back towards free catalyst. We monitored the reaction by ^{1}H NMR and found that around 30% of the catalyst was the iminium of hydrocinnamaldehyde and catalyst **3b** and the 70% remaining was *N*-brominated. We observed that the succinimide accelerated the reaction and all the NBS was consumed less than 2h, with an overall yield of 52.3% (47.5% dibrominated and 4.8% monobrominated aldehyde, Figure S14).

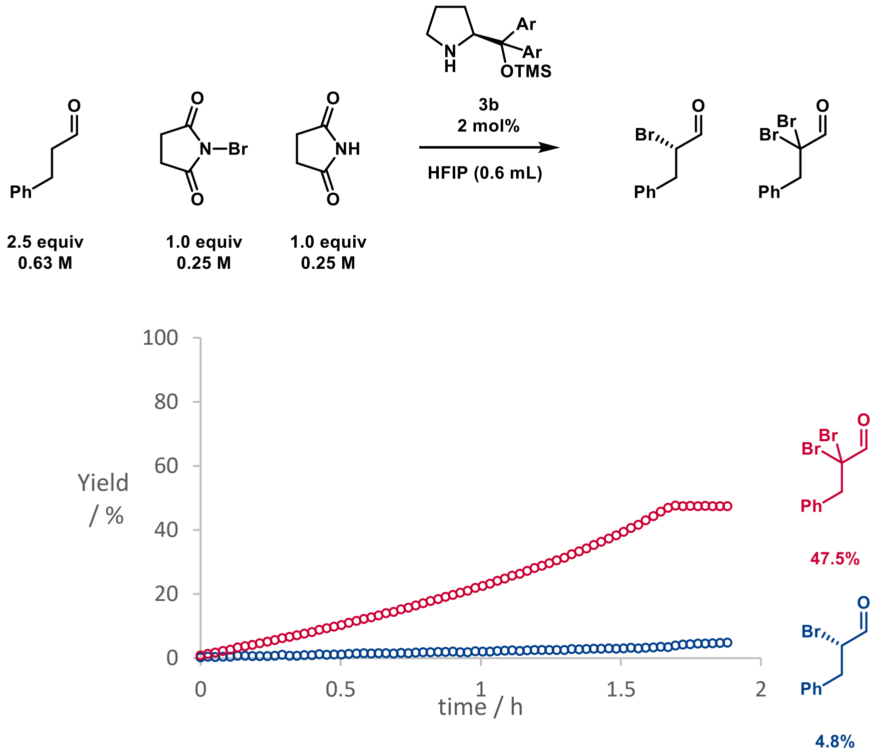

Figure S14. Adding succinimide accelerates the reaction, but most of the product is still dibrominated aldehyde.

As the product was still mostly dibrominated aldehyde (Figure S14), we repeated the reaction with added succinimide (0.15 mmol) and water (50 µL) to accelerate the hydrolysis of the bromoenamine relative to its bromination. As we expected, the addition of water reduced the amount of dibromination (15%) and gave a good yield of monobrominated aldehyde (73%, Figure S15).

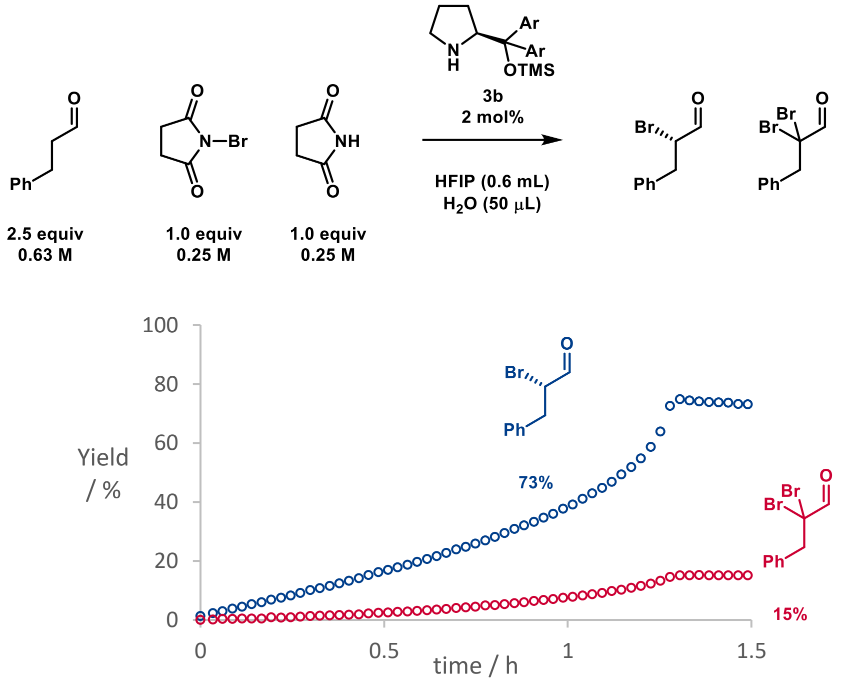

Figure S15. Adding water and succinimide to the reaction provides a good yield of monobrominated aldehyde in less than 90 min.

However, when we checked the enantiomeric excess of the monobrominated aldehyde from this reaction, we found it to be very disappointing (78:22). We hypothesized that pathways involving the brominated catalyst acting as the brominating agent or succinimide/catalyst-induced product racemization may have contributed to the poor enantioselectivity.

Chiralpak OD-H, 4.6 x 250 mm, 220 nm detection, 1 mL/min Hexane:IPA 97:3

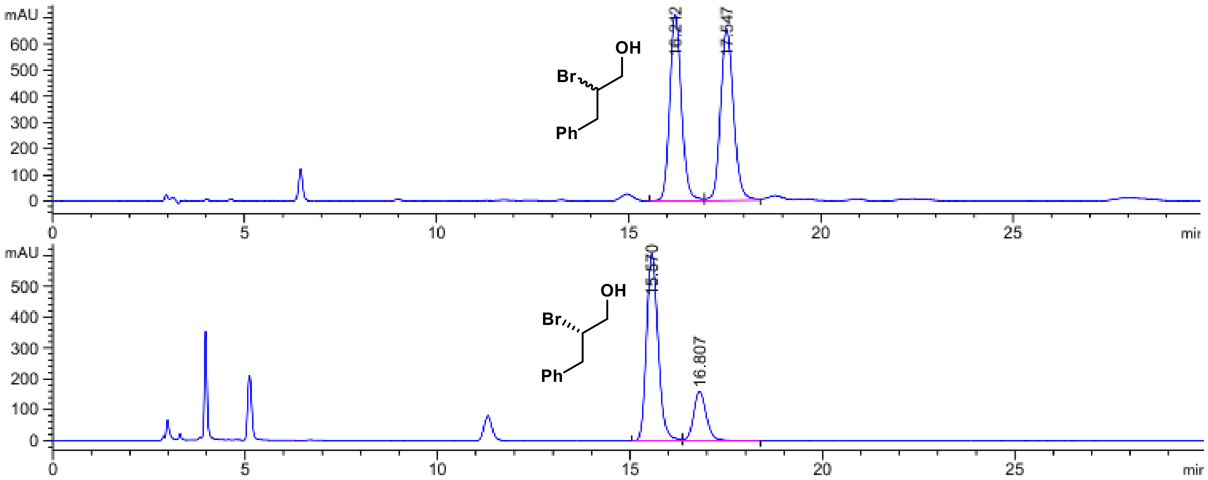

Peak #	RetTime [min]	Type	Width [min]	Area [mAU*s]	Height [mAU]	Area %
1	15.570	BV	0.3286	1.28524e4	608.01990	77.7610
2	16.807	VB	0.3525	3675.67480	159.72247	22.2390

4.4 Uncatalyzed Reaction

We appreciated the recent report of H-bonding between NBS and HFIP to give a more active brominating agent.[7] We considered the possibility that this activated brominating agent could allow an uncatalyzed background reaction between the aldehyde and NBS and potentially reduce the overall enantiomeric excess of the brominated products. We mixed NBS (0.12 mmol) and pentanal (0.12 mmol) in HFIP (0.6 mL) at room temperature and monitored the reaction by ^{1}H NMR. We compared the integrals of the aldehyde protons on the starting material (9.72 ppm), mono- (9.43 ppm) and dibrominated aldehydes (9.26 ppm).

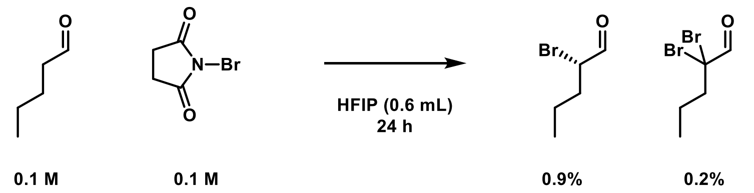

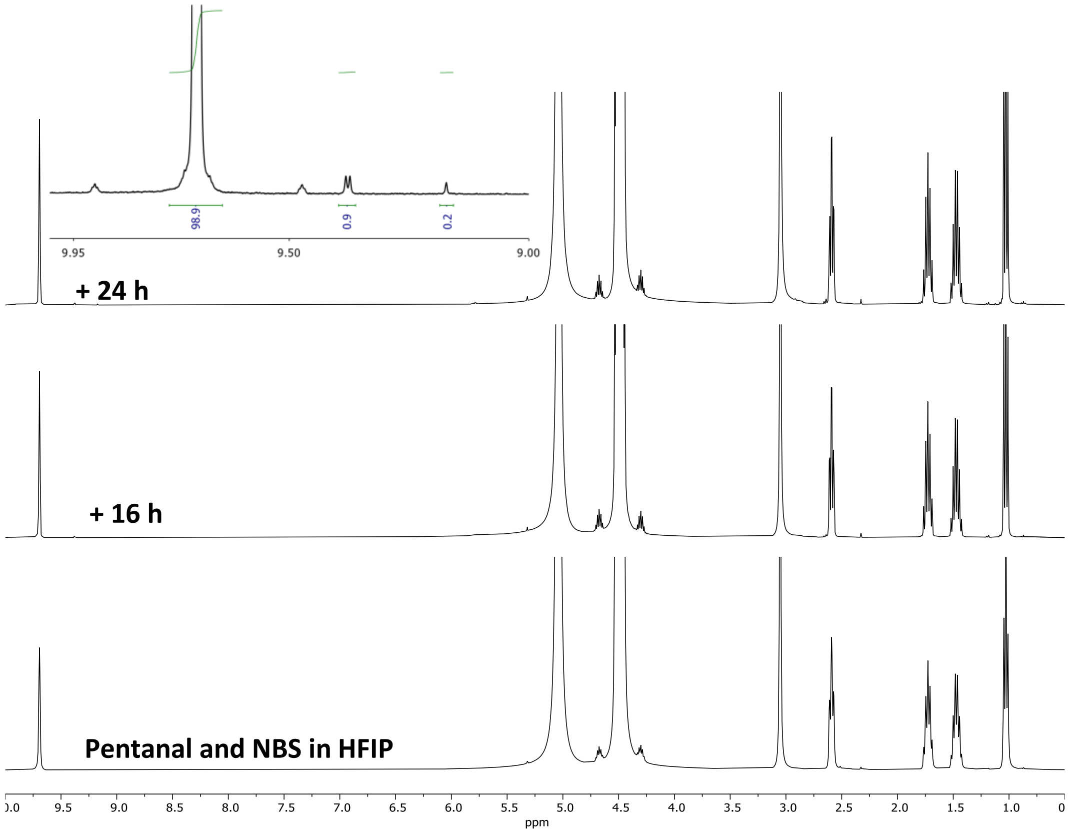

Figure S16. Very little background reaction between NBS and pentanal occurred over 24 h at room temperature

We observed very low conversion to the monobrominated (0.9%) and dibrominated (0.2%) aldehydes after 24 h, based on the relative integrals of the aldehyde signals (Figure S16). This suggested that background reactions would not make significant contributions to the outcomes of our brominations.

4.5 Bromination of propanal

We screened several initial amounts of water for our enantioselective, aminocatalytic α-bromination of propanal following **GP2** with an addition time of 150 min. We selected propanal as it was the most challenging substrate in our scope and did not provide a good enantiomeric excess. We measured the yield by ^1H qNMR with an internal standard (DNB) after reduction and the enantiomeric excess after derivatization of the α-brominated aldehyde to the benzoylated-alcohol analogue.

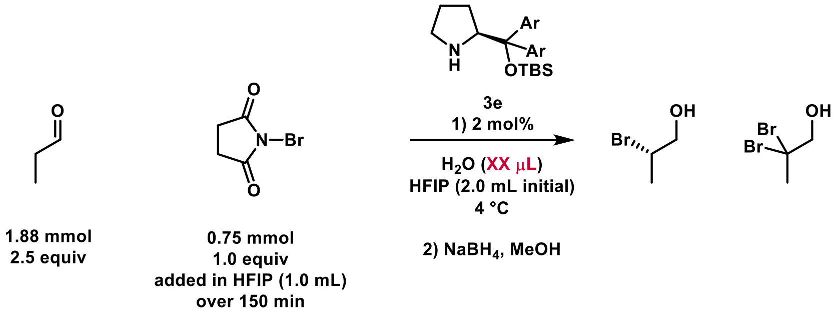

exp	water / µL	monobrominated product / %	dibrominated product / %	er
**1**	50	23	30	88:12
**2**	100	35	32	86:14
**3**	150	42	29	84:16
**4**	200	61	22	76:24

Increasing the initial amount of water increased the yield of monobrominated aldehyde and reduced the enantioselectivity of the reaction. However, even when there is more dibrominated aldehyde than monobrominated, the enantiomeric ratio did not exceed 88:12. We hypothesized that these results indicate that dibromination does not act as an effective resolution for this substrate or that 2-bromo-propanal can racemize more quickly than other substrates.

4.6 Enantiomeric ratio over the slow addition

We ran three different α-brominations of pentanal following **GP2** with a 75 min addition to see if the enantiomeric ratio of the product changed over the slow addition. We selected pentanal because it displayed a poor enantiomeric excess like propanal but is not as volatile. The first reaction was quenched after 25 min (33% addition), the second after 50 min (67% addition) and the third one after the complete addition of NBS. We calculated the NBS consumption and ratio of mono:dibromination using qNMR with an internal standard (NTB). We also measured the enantiomeric excess of the monobrominated product in the samples after reduction and derivatization of the α-brominated aldehyde to the corresponding benzoylated-alcohol analogue.

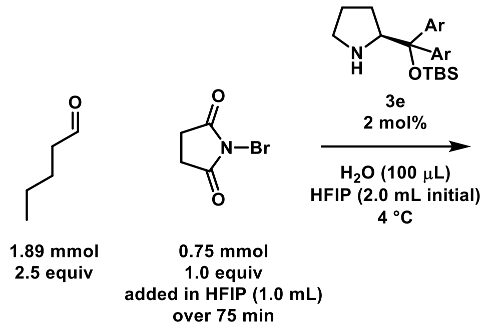

sample	NBS added / %	NBS consumed / %	mono:dibromination	er
1	33	32	1:0.45	91:9
2	67	66	1:0.45	90:10
3	100	100	1:0.48	89:11

We found that the ratio of dibromination increased slightly over the reaction course and that the enantiomeric excess falls slightly. These results are consistent with our observation that the product loses enantiomeric excess in the reaction mixture but does not explain the lower-than-expected enantioselectivities for this substrate or other short-chain substrates.

5. Optimization tables

We have documented the reactions we ran with each substrate to optimize the time of addition and amount of added water.

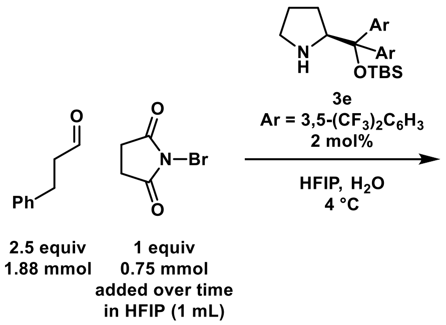

exp	time / min	water / µL	monobrominated product / %	dibrominated product / %	er
**1**	50	25	50	16	--
**2**	60	50	71	15	98:2

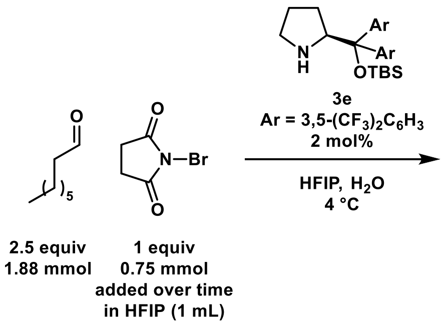

exp	time / min	water / µL	monobrominated product / %	dibrominated product / %	er
**1**	60	40	35	32	--
**2**	60	80	73	11	95:5

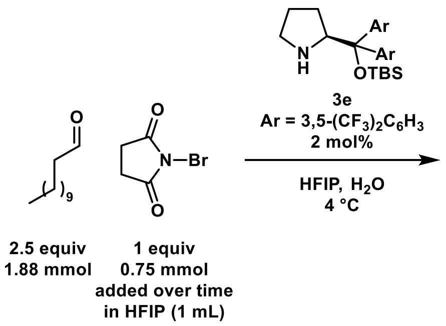

exp	time / min	water / µL	monobrominated product / %	dibrominated product / %	er
**1**	60	80	44	24	96:4

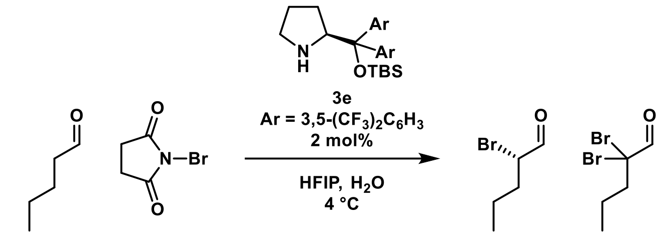

2.5 equiv 1.88 mmol

1 equiv 0.75 mmol added over time in HFIP (1 mL)

exp	time / min	water / µL	monobrominated product / %	dibrominated product / %	er
**1**	75	80	45	28	90:10
**2**	75	100	65	17	92:8
**3**	75	200	80	10	87:13

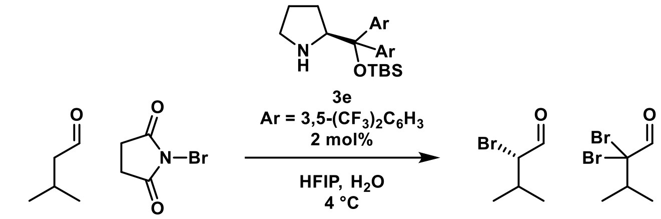

2.5 equiv 1.88 mmol

1 equiv 0.75 mmol added over time in HFIP (1 mL)

exp	time / min	water / µL	monobrominated product / %	dibrominated product / %	er
**1**	240	50	21	22	--
**2**	285	75	51	25	--
**3**	285	85	69	15	95:5

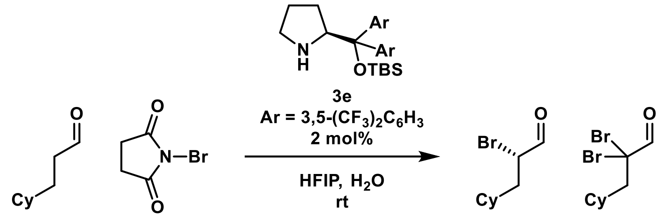

2.5 equiv 1.88 mmol

1 equiv 0.75 mmol added over time in HFIP (1 mL)

exp	time / min	water / µL	monobrominated product / %	dibrominated product / %	er
**1** (4 °C)	60	70	--	--	--
**2** (4 °C)	285	70	--	--	--
**3**	60	70	80	10	87:13
**4**	90	50	72	12	90:10

6. NMR Spectra of Brominated Products

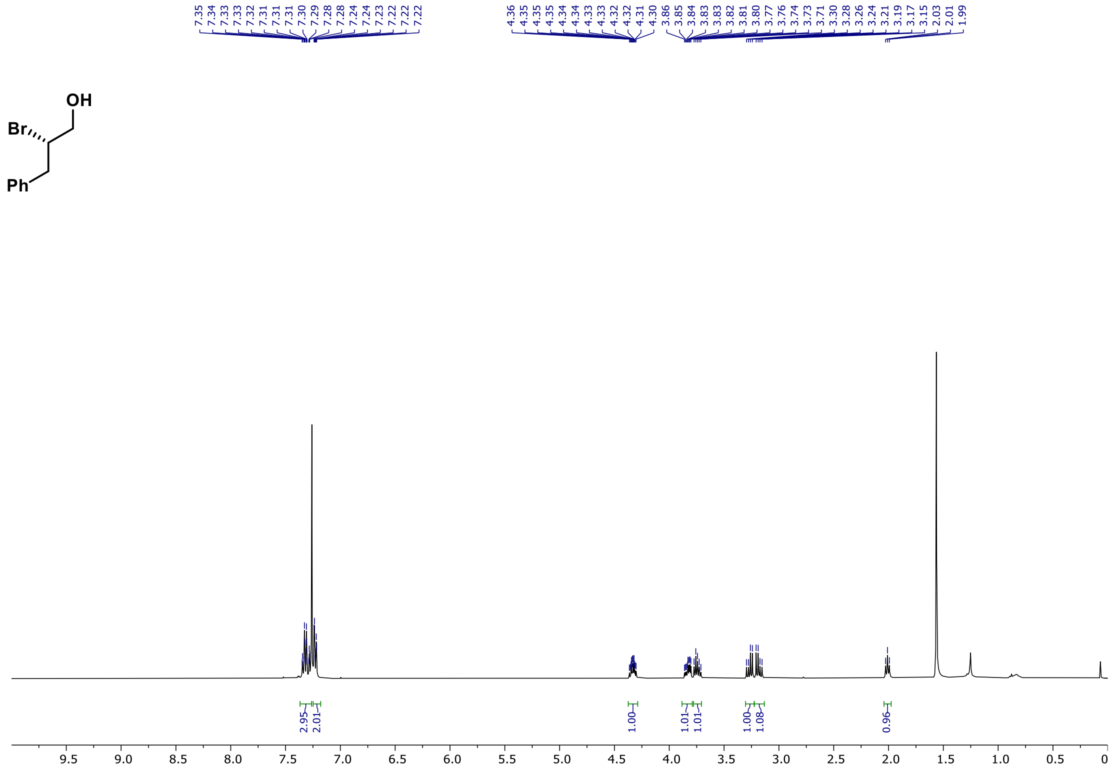

Figure S17. ^{1}H NMR (400 MHz, $CDCl_3$) spectrum of 2-bromo-3-phenylpropan-1-ol

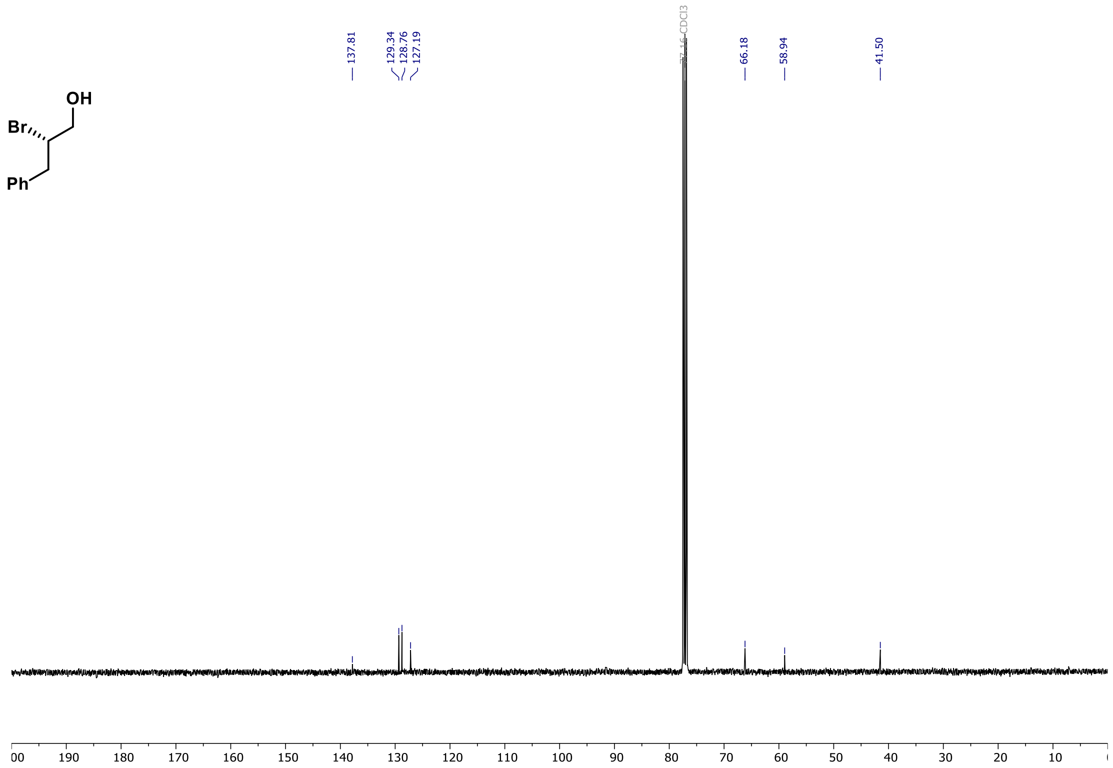

Figure S18. $^{13}C\{^{1}H\}$ NMR (101 MHz, $CDCl_3$) spectrum of 2-bromo-3-phenylpropan-1-ol

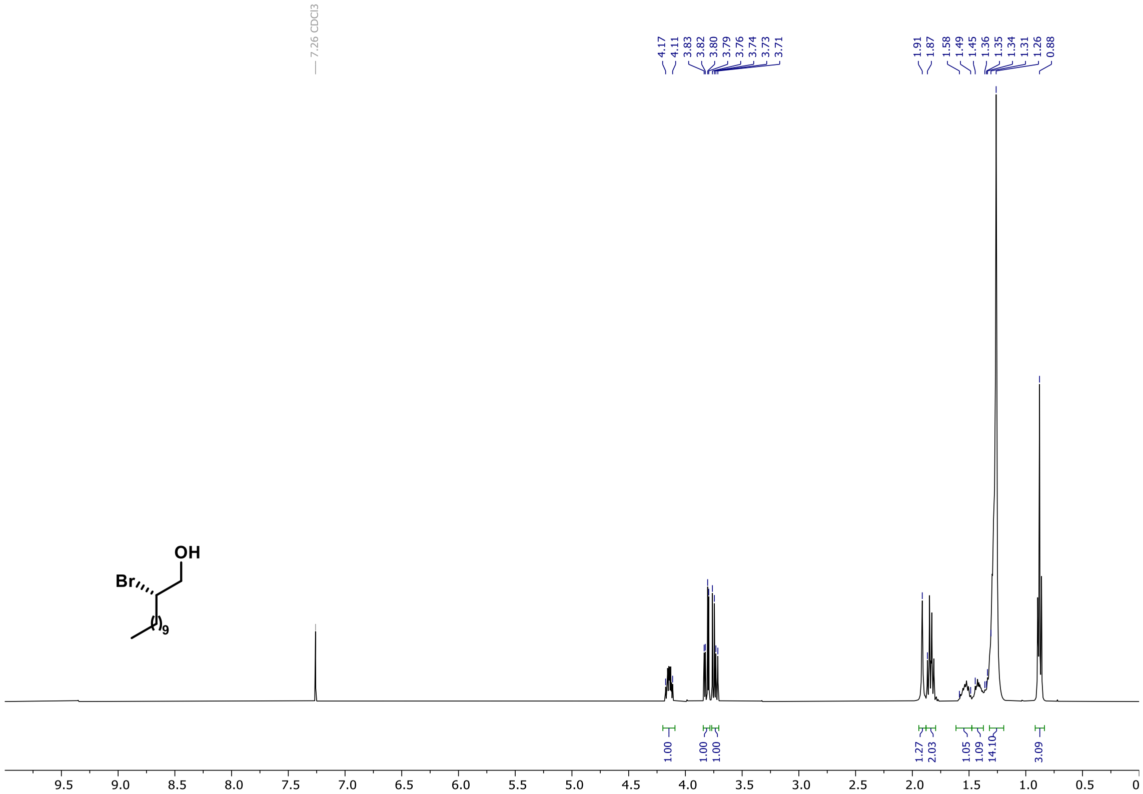

Figure S19. ¹H NMR (400 MHz, $CDCl_3$) spectrum of 2-bromo-dodecan-1-ol

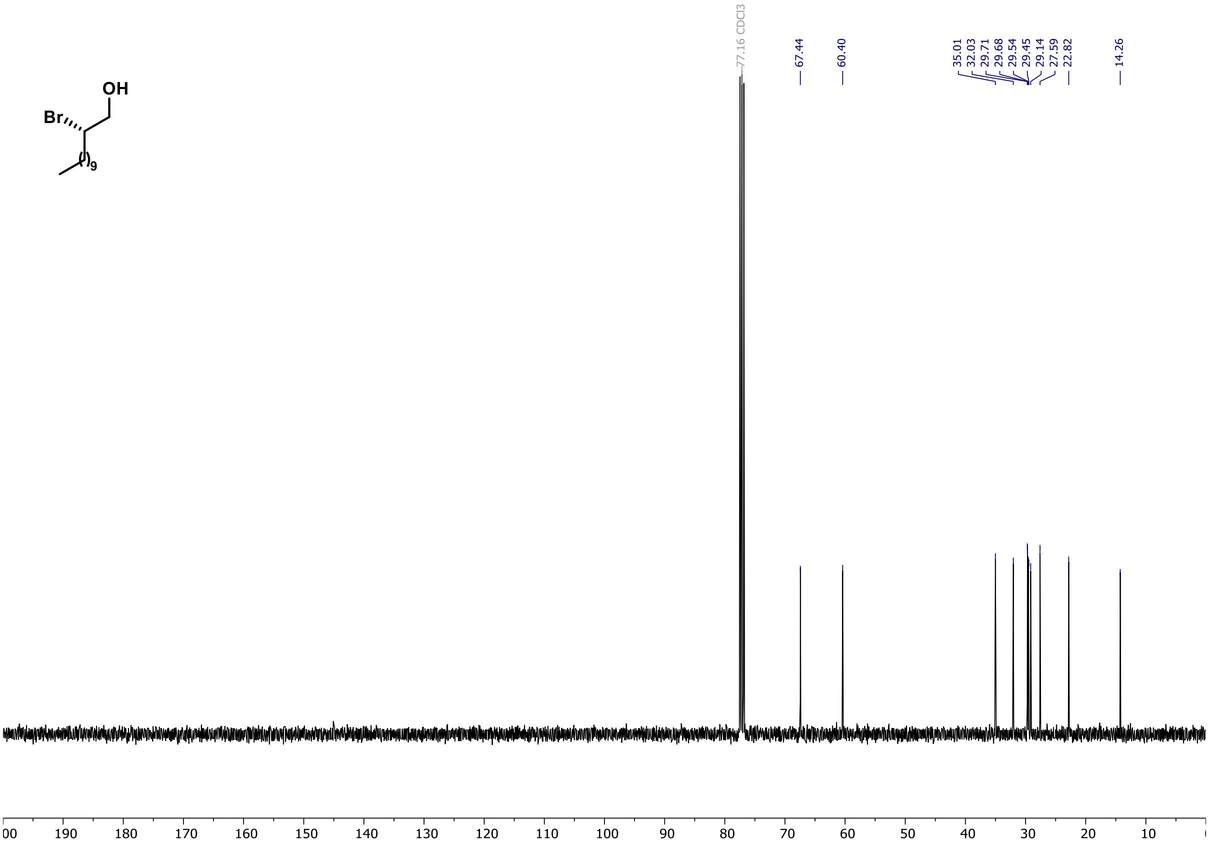

Figure S20. $^{13}C\{^1H\}$ NMR (101 MHz, $CDCl_3$) spectrum of 2-bromo-dodecan-1-ol

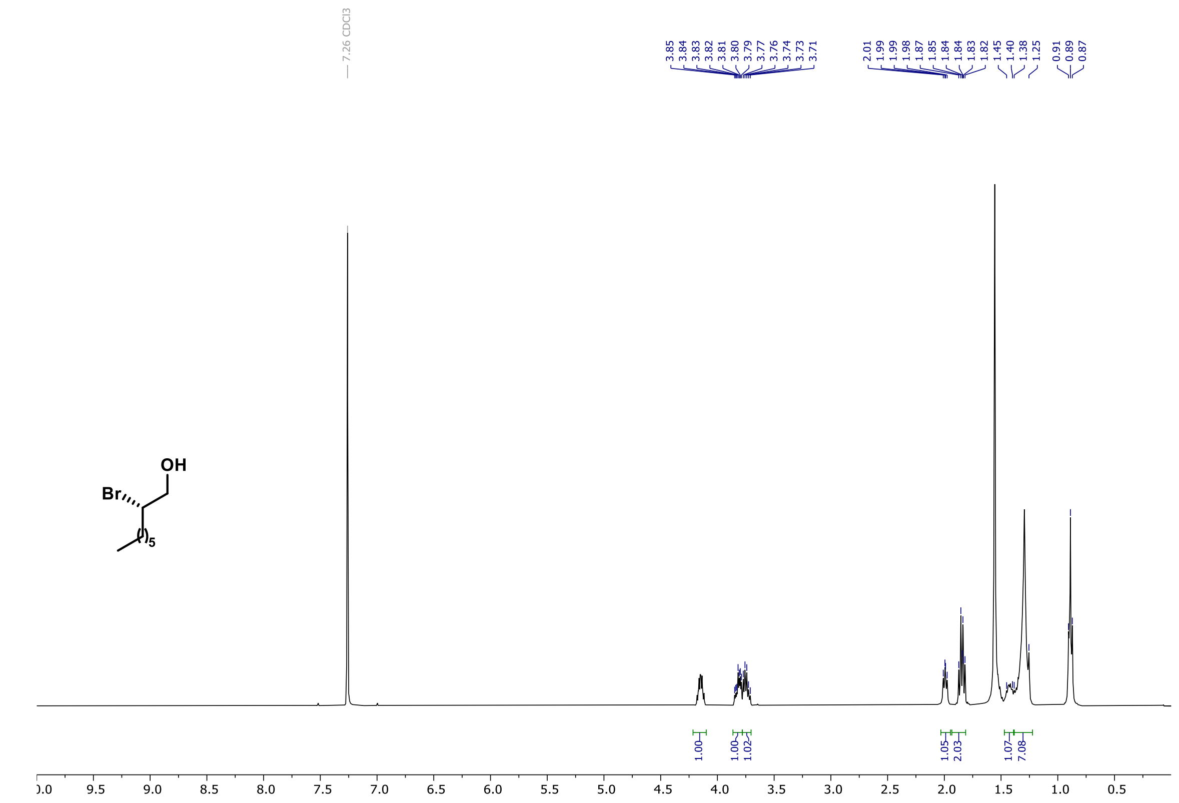

Figure S21. ^{1}H NMR (400 MHz, $CDCl_3$) spectrum of 2-bromo-octan-1-ol

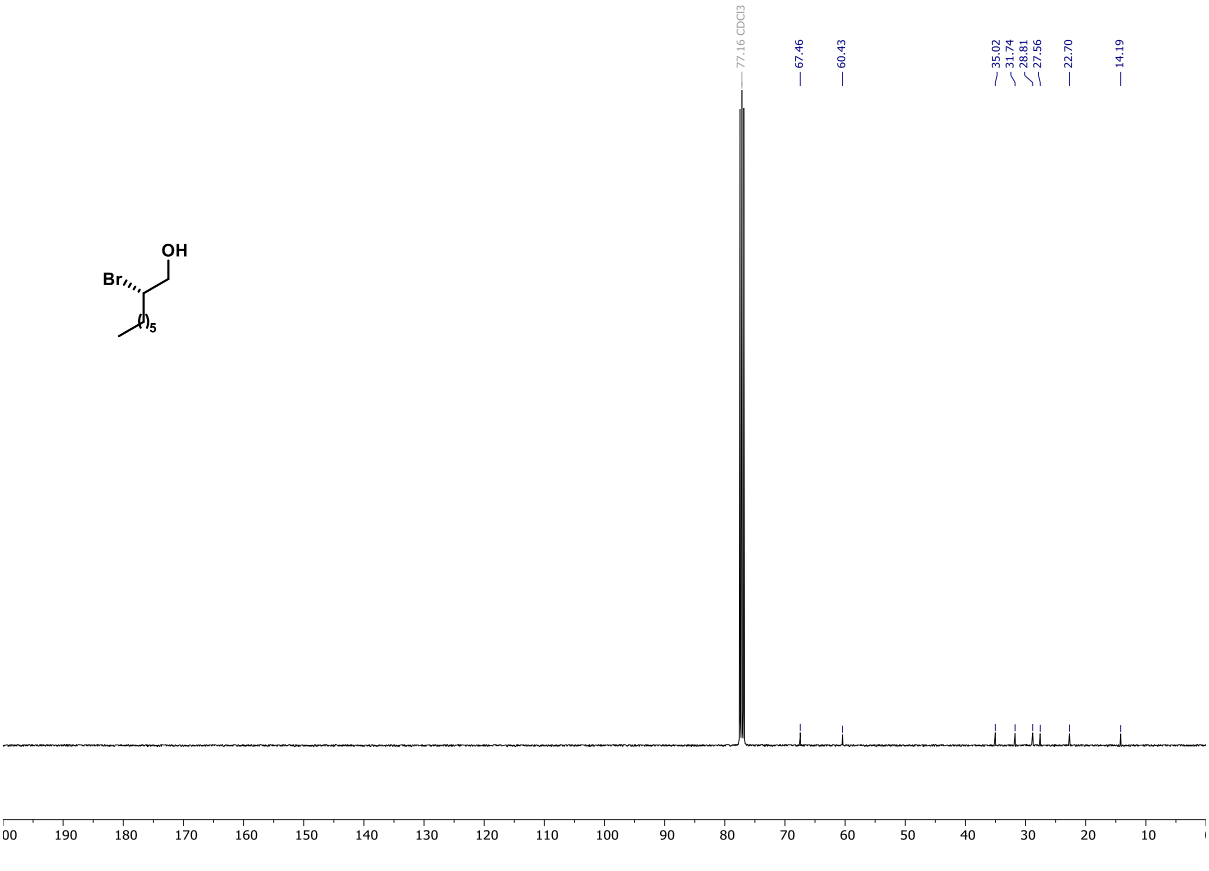

Figure S22. $^{13}C\{^{1}H\}$ NMR (101 MHz, $CDCl_3$) spectrum of 2-bromo-octan-1-ol

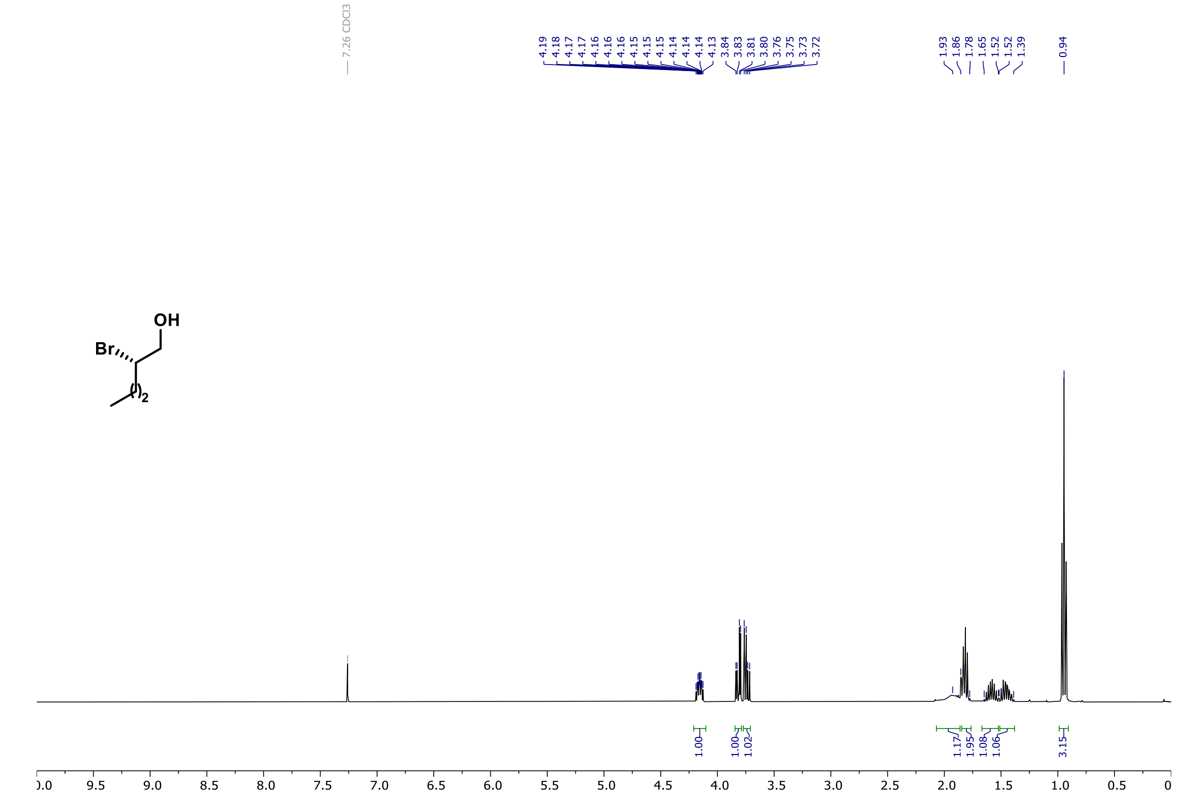

Figure S23. ^{1}H NMR (400 MHz, $CDCl_3$) spectrum of 2-bromo-pentan-1-ol

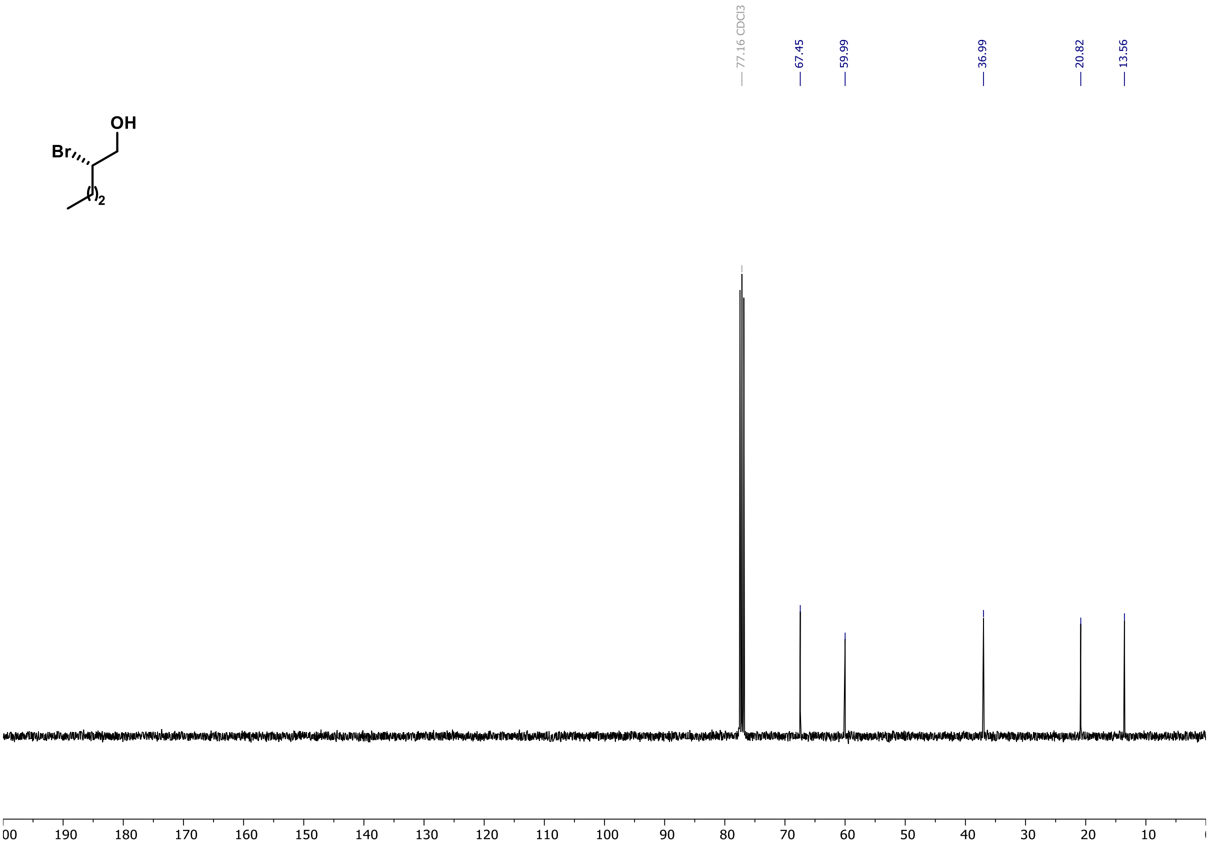

Figure S24. $^{13}C\{^{1}H\}$ NMR (101 MHz, $CDCl_3$) spectrum of 2-bromo-pentan-1-ol

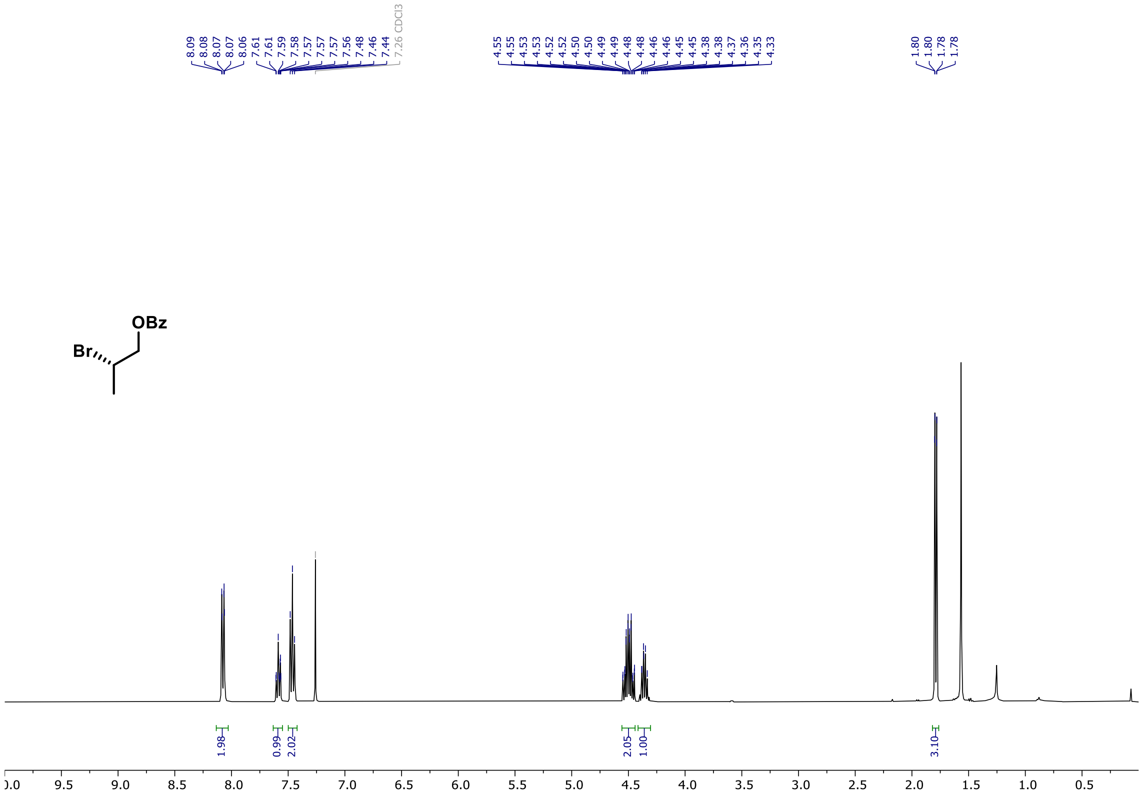

Figure S25. ^{1}H NMR (400 MHz, $CDCl_3$) spectrum of 2-bromopropylbenzoate

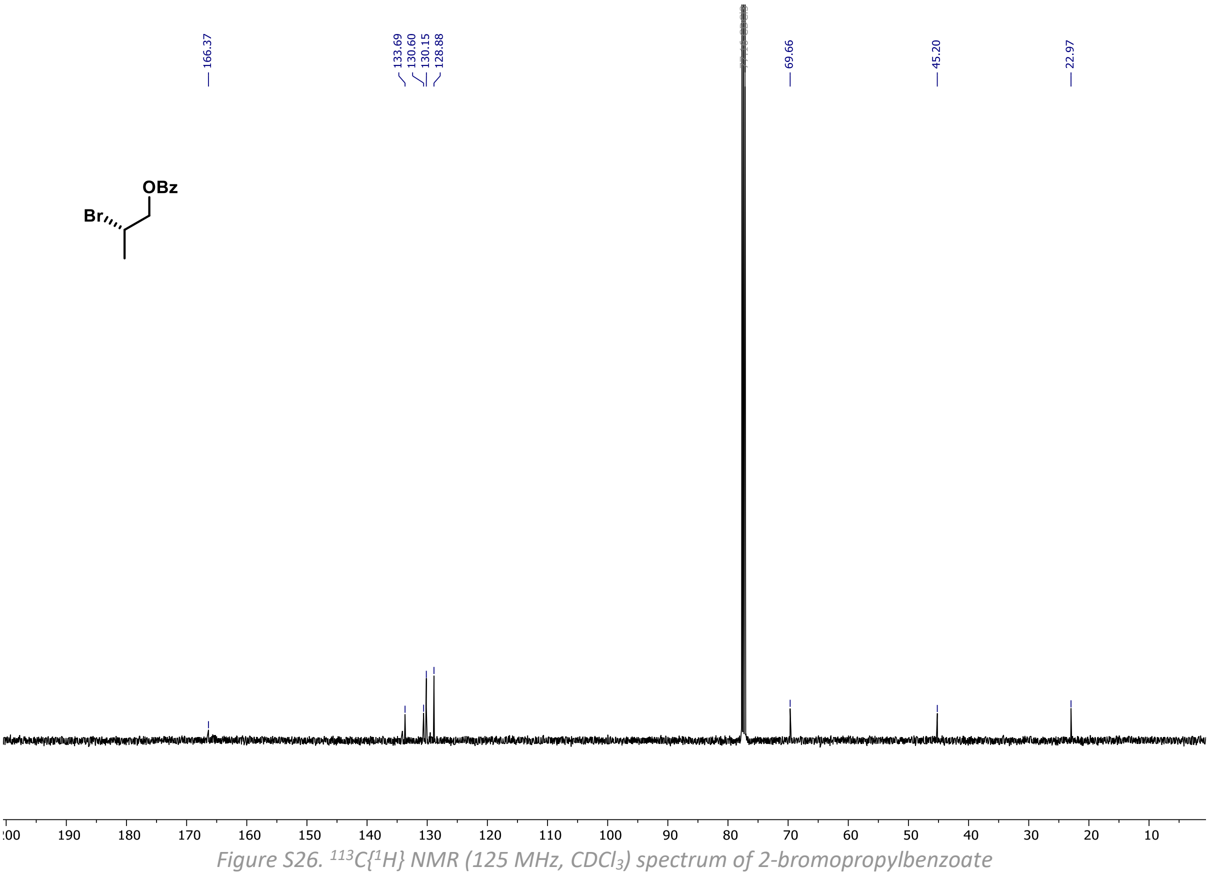

Figure S26. $^{113}C\{^{1}H\}$ NMR (125 MHz, $CDCl_3$) spectrum of 2-bromopropylbenzoate

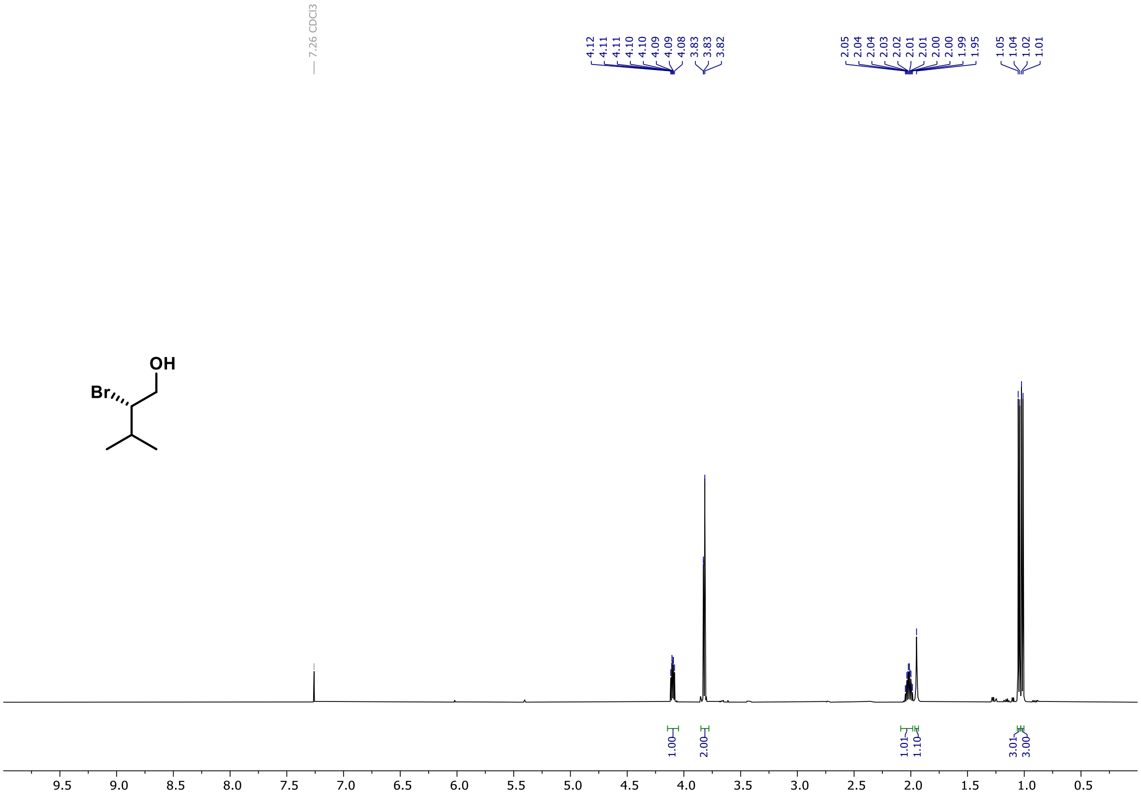

Figure S27. ¹H NMR (500 MHz, $CDCl_3$) spectrum of 2-bromo-3-methylbutan-1-ol

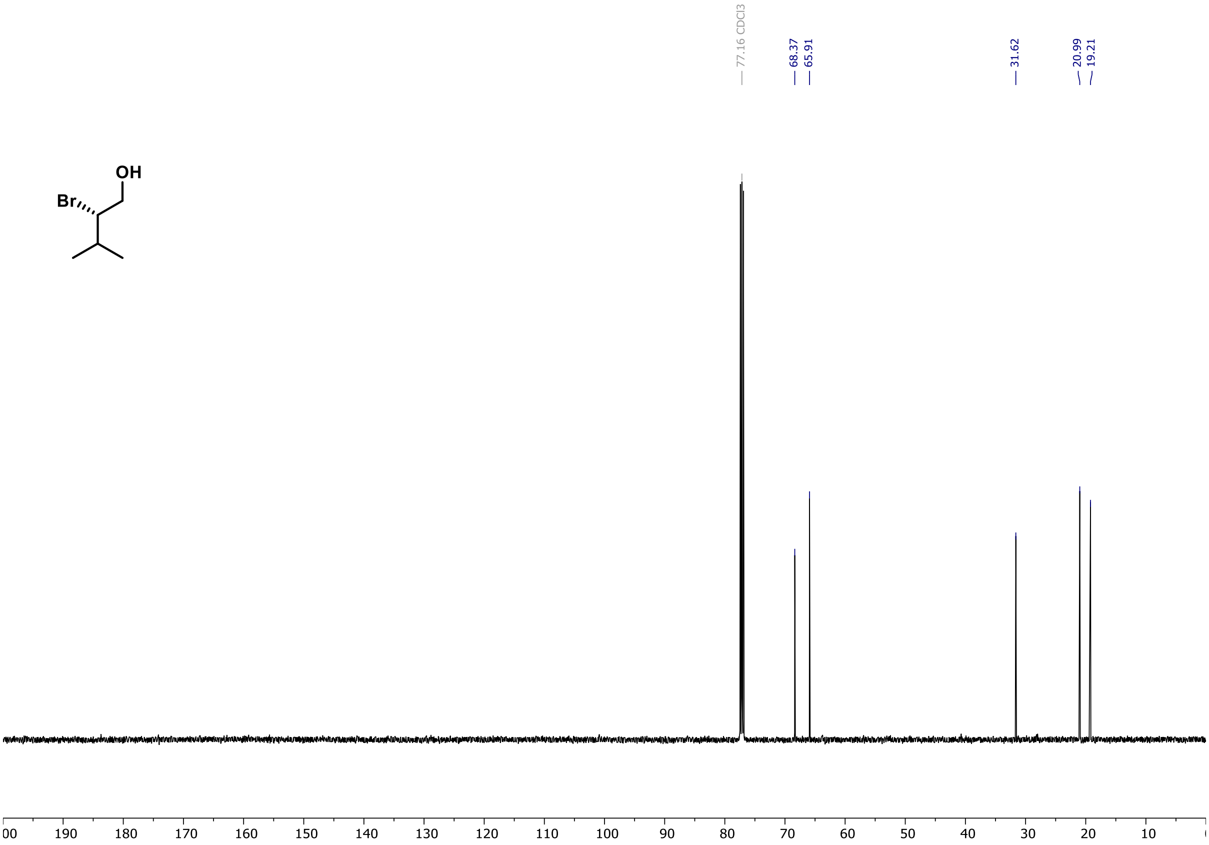

Figure S28. $^{13}C\{^1H\}$ NMR (125 MHz, $CDCl_3$) spectrum of 2-bromo-3-methylbutan-1-ol

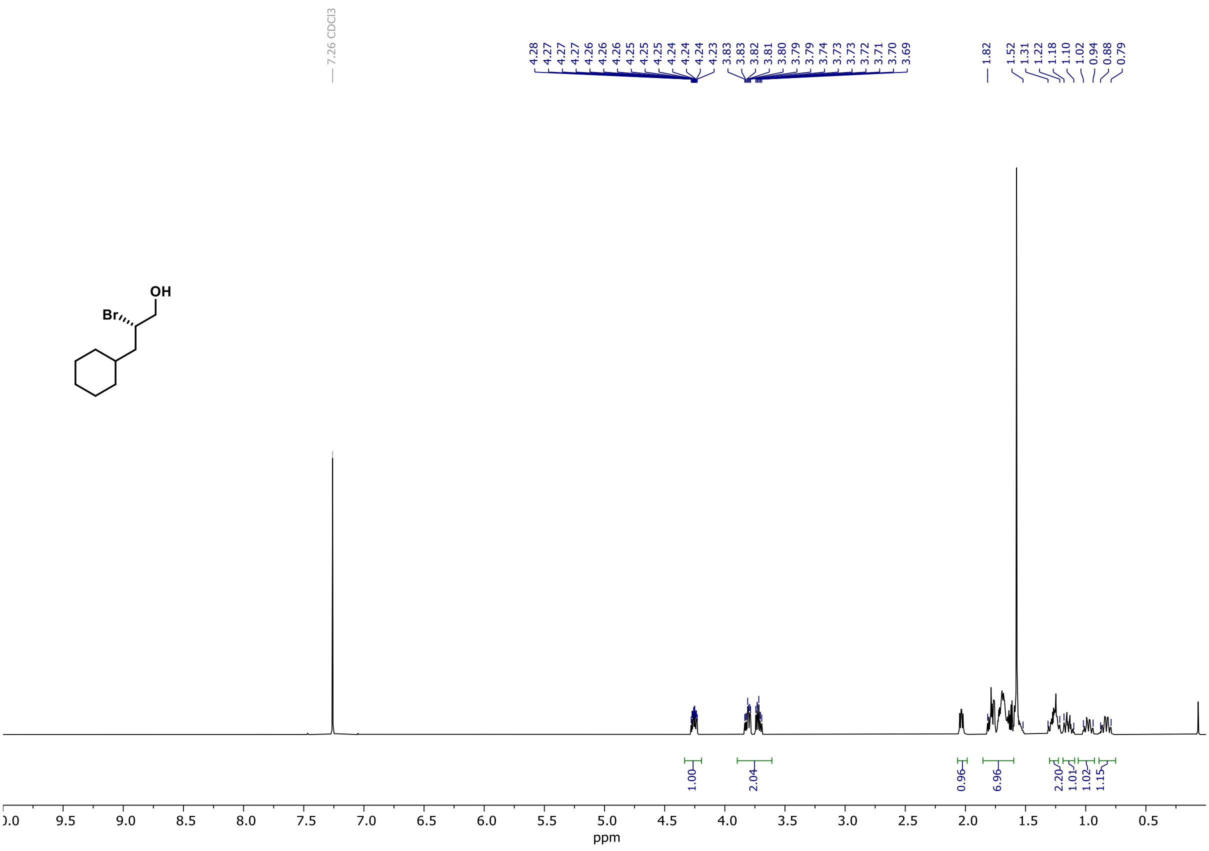

Figure S29. ^{1}H NMR (500 MHz, $CDCl_3$) spectrum of 2-bromo-3-cyclohexylpropan-1-ol

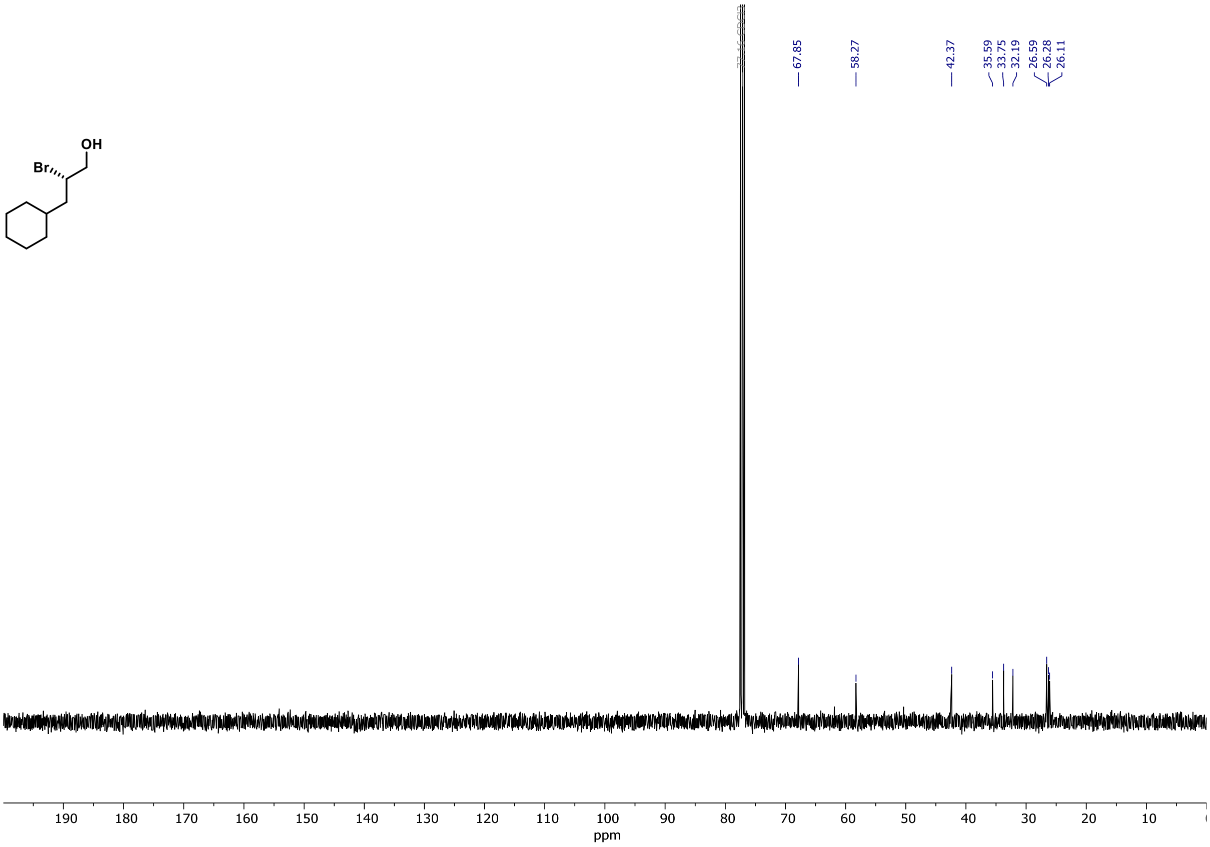

Figure S30. $^{13}C\{^{1}H\}$ NMR (125 MHz, $CDCl_3$) spectrum of 2-bromo-3-cyclohexylpropan-1-ol.

7. Determination of the enantiomeric ratio of the brominated products

2-Bromo-3-phenylpropan-1-ol

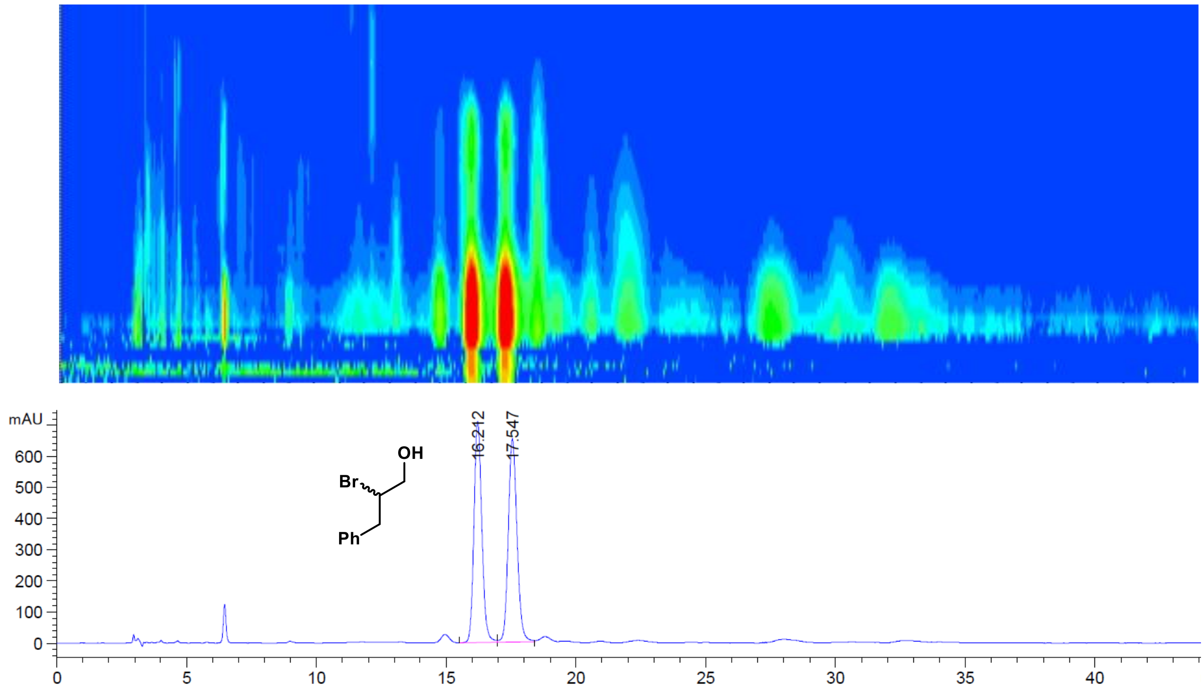

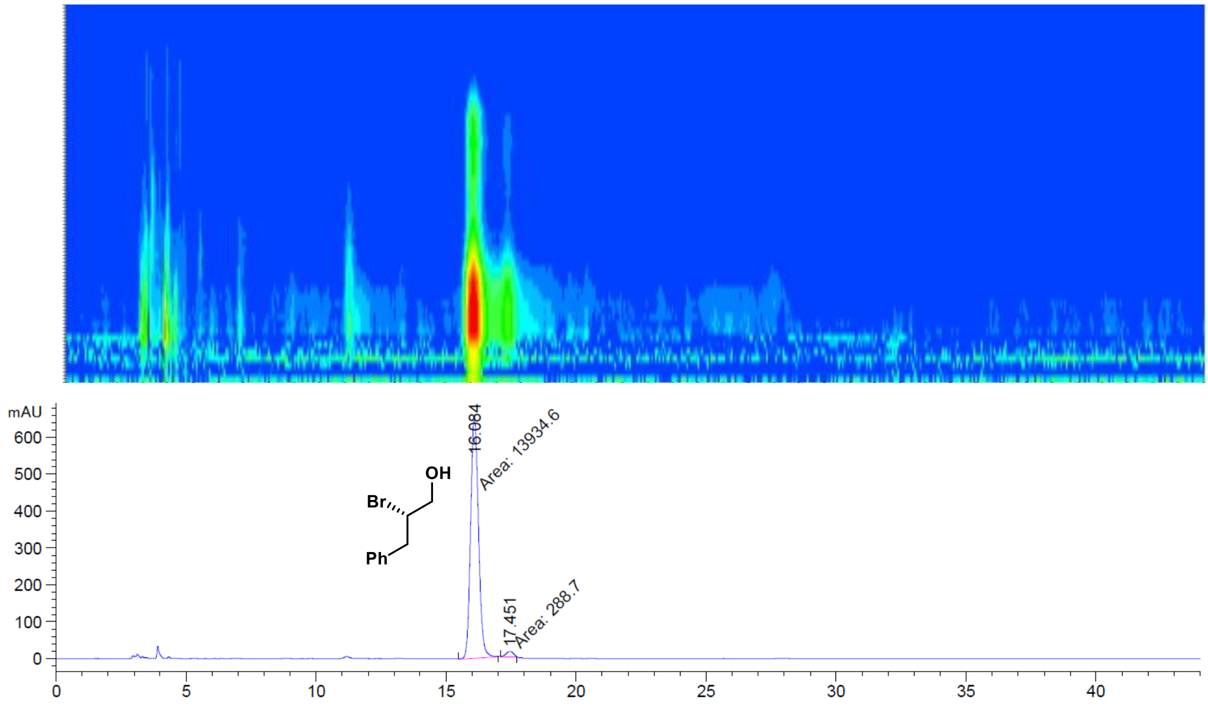

Peak #	RetTime [min]	Type	Width [min]	Area [mAU*s]	Height [mAU]	Area %
1	16.084	MM	0.3527	1.39346e4	658.49664	97.9702
2	17.451	MM	0.3255	288.70016	14.78375	2.0298

Chiralpak OD-H, 4.6 x 250 mm, 208 nm detection, 1 mL/min Hexane:IPA 97:3

2-Bromo-3-phenylpropan-1-ol (3 mmol scale)

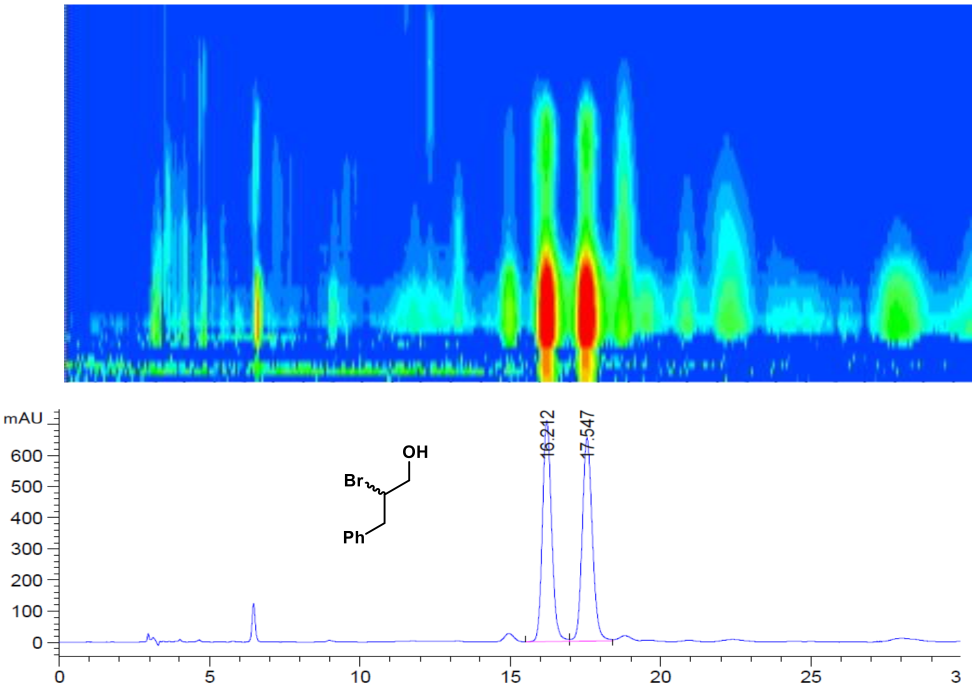

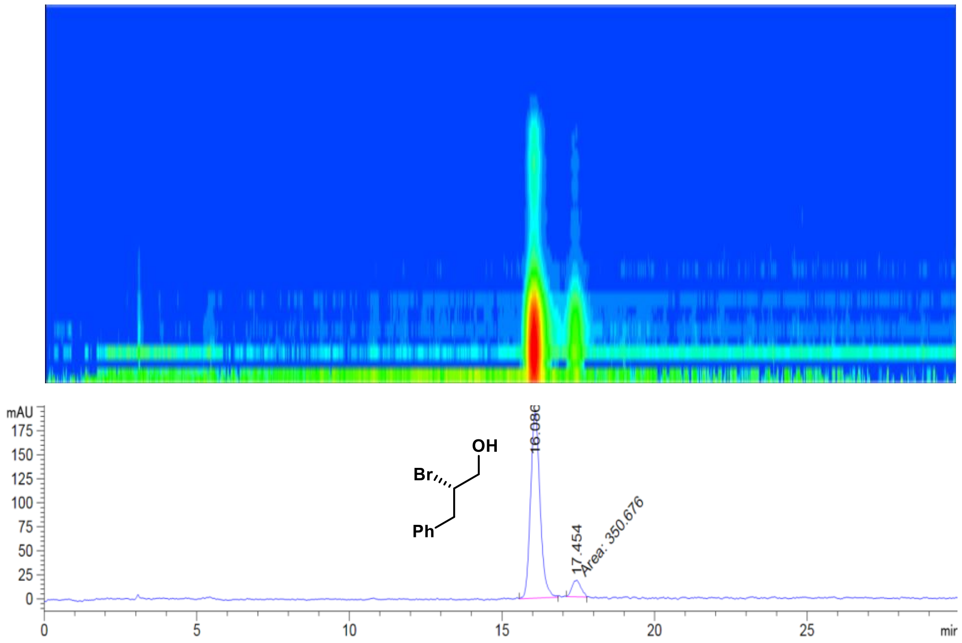

Peak #	RetTime [min]	Type	Width [min]	Area [mAU*s]	Height [mAU]	Area %
1	16.086	VV	0.3233	4165.81689	193.40967	92.2357
2	17.454	MM	0.3405	350.67609	17.16296	7.7643

Chiralpak OD-H, 4.6 x 250 mm, 208 nm detection, 1 mL/min Hexane:IPA 97:3

2-Bromo-dodecan-1-ol

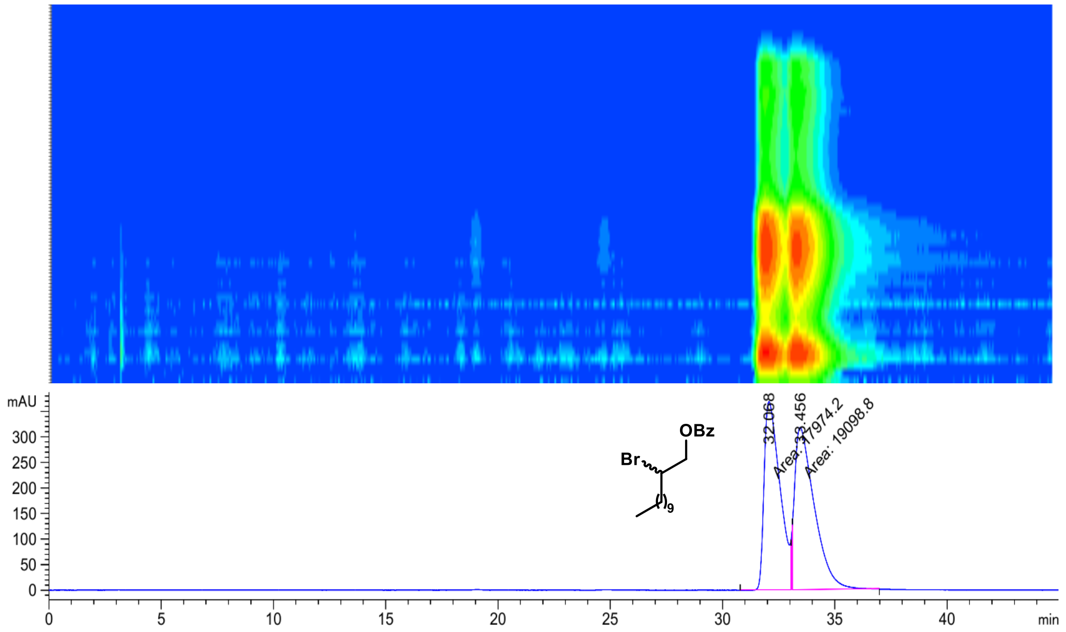

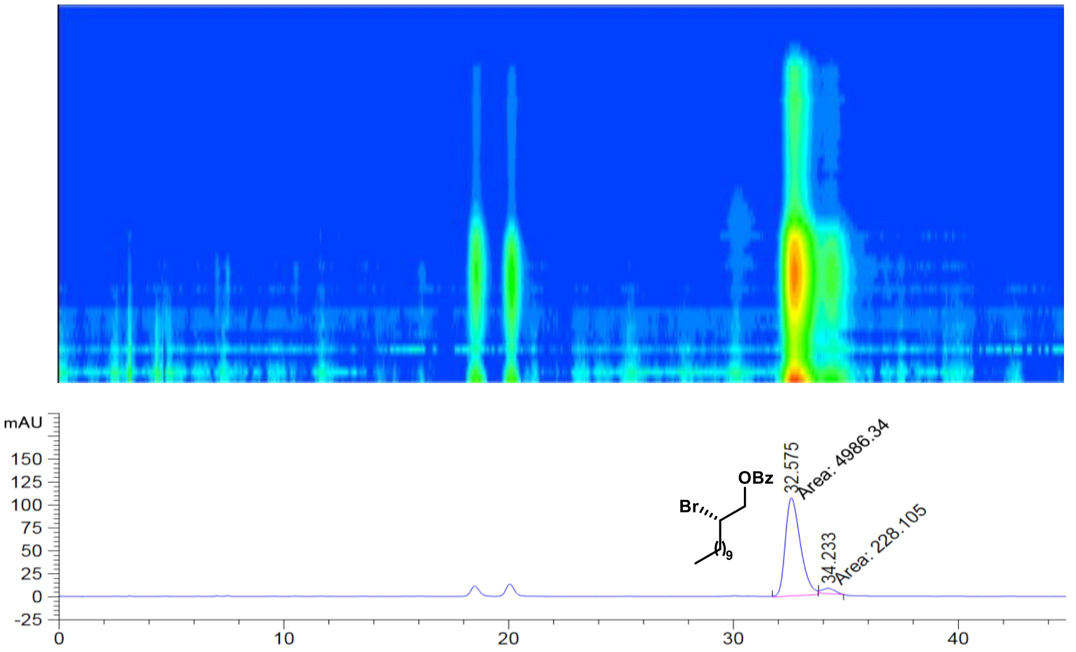

Peak #	RetTime [min]	Type	Width [min]	Area [mAU*s]	Height [mAU]	Area %
1	32.575	MM	0.7779	4986.33789	106.83965	95.6255
2	34.233	MM	0.6703	228.10490	5.67176	4.3745

Chiralpak OD-H, 4.6 x 250 mm, 230 nm detection, 1 mL/min Hexane:IPA 99:1

2-Bromo-octan-1-ol

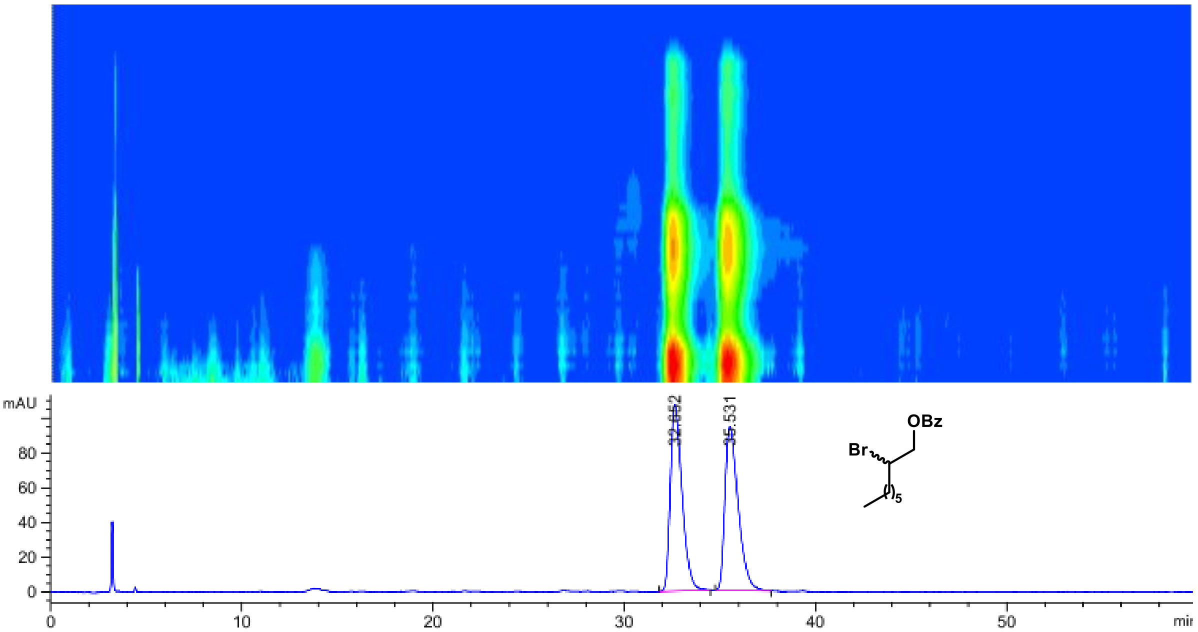

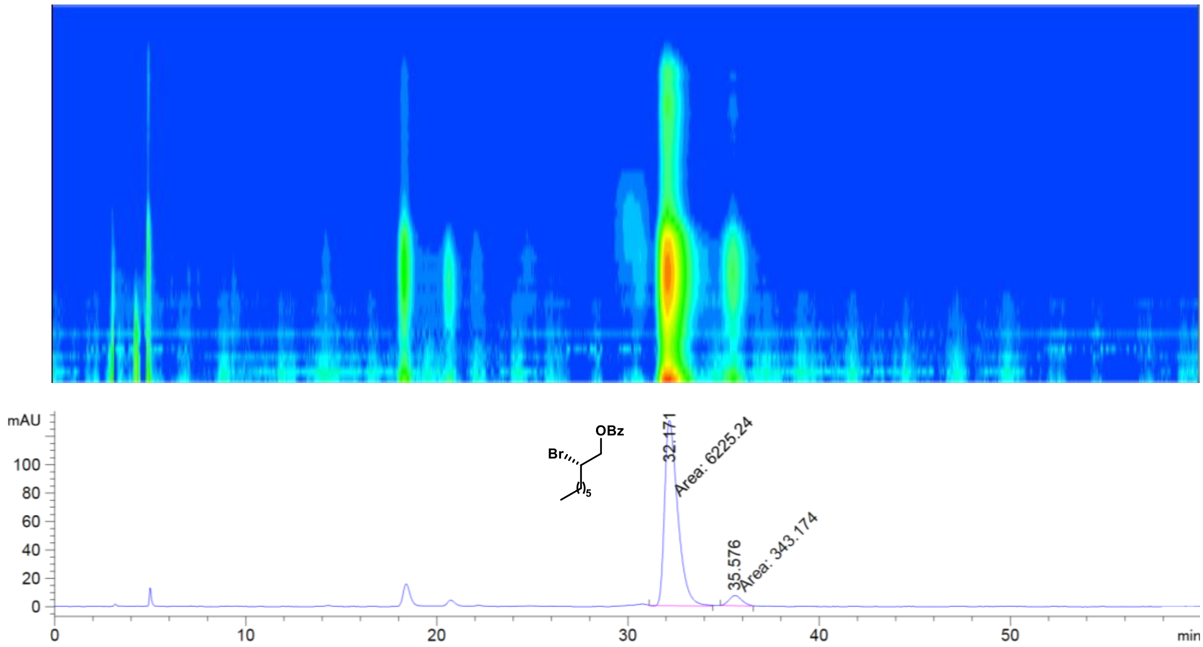

Peak #	RetTime [min]	Type	Width [min]	Area [mAU*s]	Height [mAU]	Area %
1	32.171	MM	0.7950	6225.24463	130.51556	94.7754
2	35.576	MM	0.7841	343.17358	7.29443	5.2246

Chiralpak OD-H, 4.6 x 250 mm, 220 nm detection, 1 mL/min Hexane:IPA 99:1

2-Bromo-pentan-1-ol

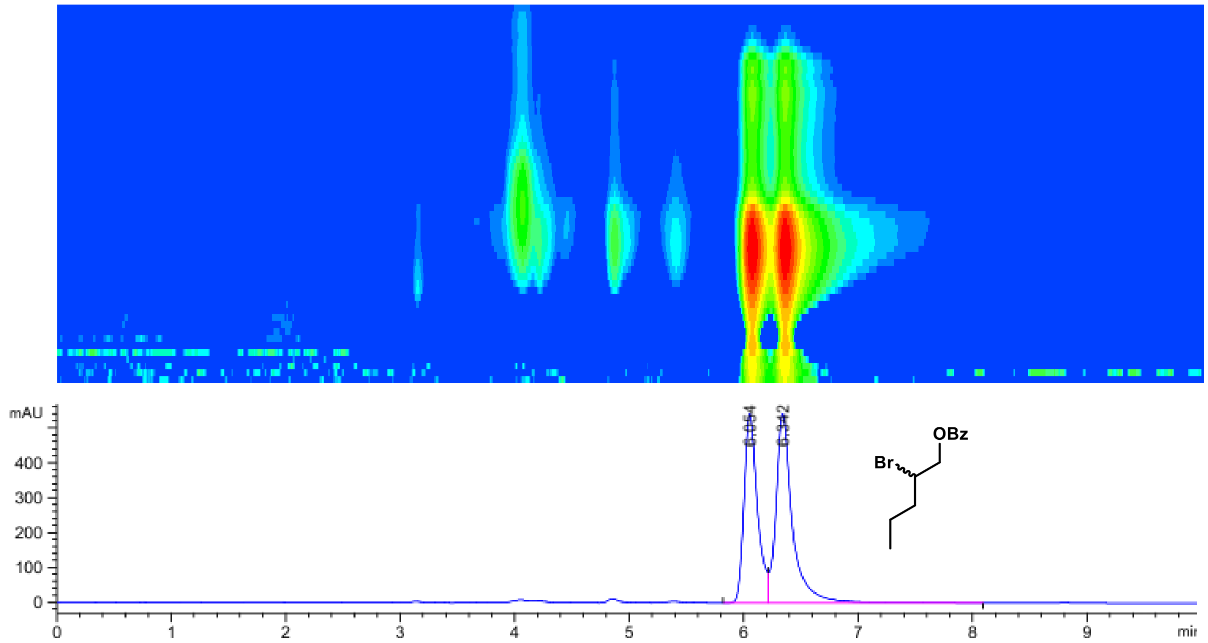

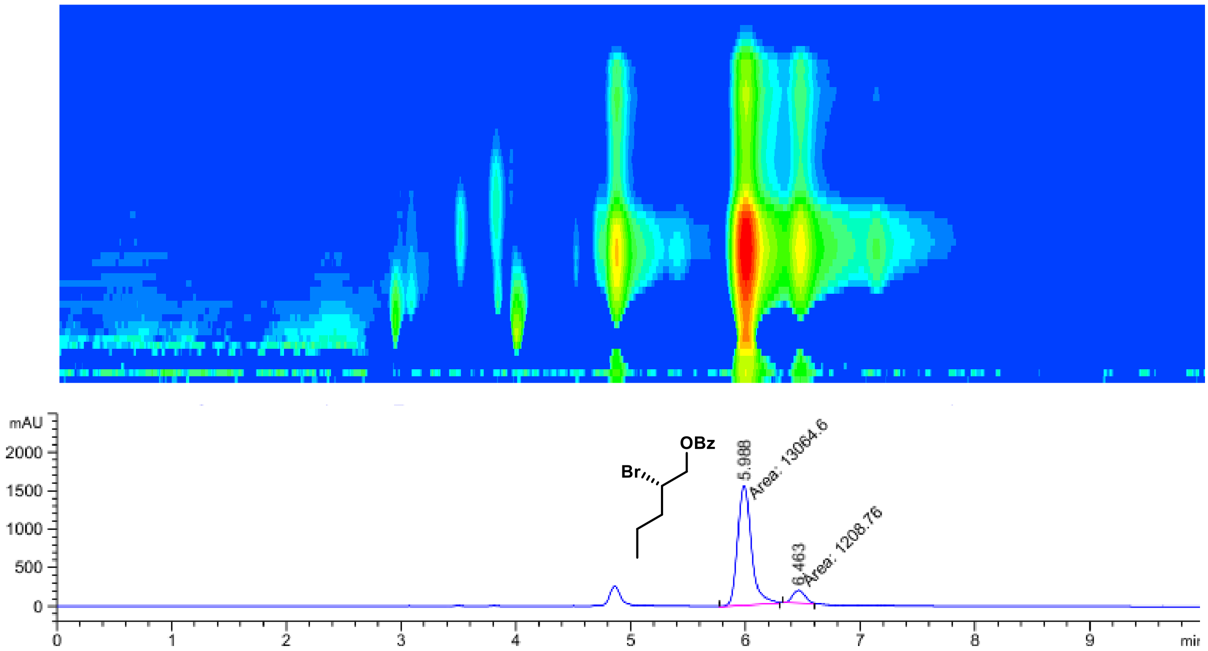

Peak #	RetTime [min]	Type	Width [min]	Area [mAU*s]	Height [mAU]	Area %
1	5.988	MM	0.1401	1.30646e4	1554.03491	91.5314
2	6.463	MM	0.1221	1208.75854	165.03564	8.4686

Chiralpak OD-H, 4.6 x 250 mm, 230 nm detection, 1 mL/min Hexane:IPA 98:2 to 98.5:1.5

2-Bromopropylbenzoate

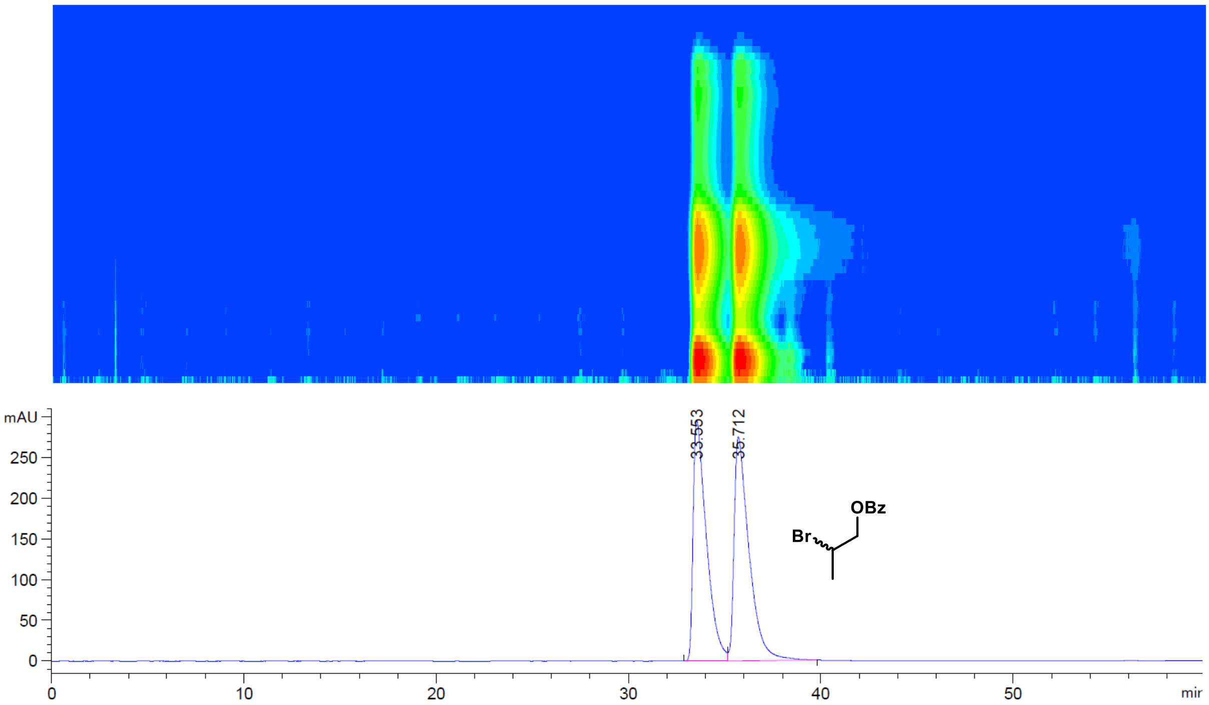

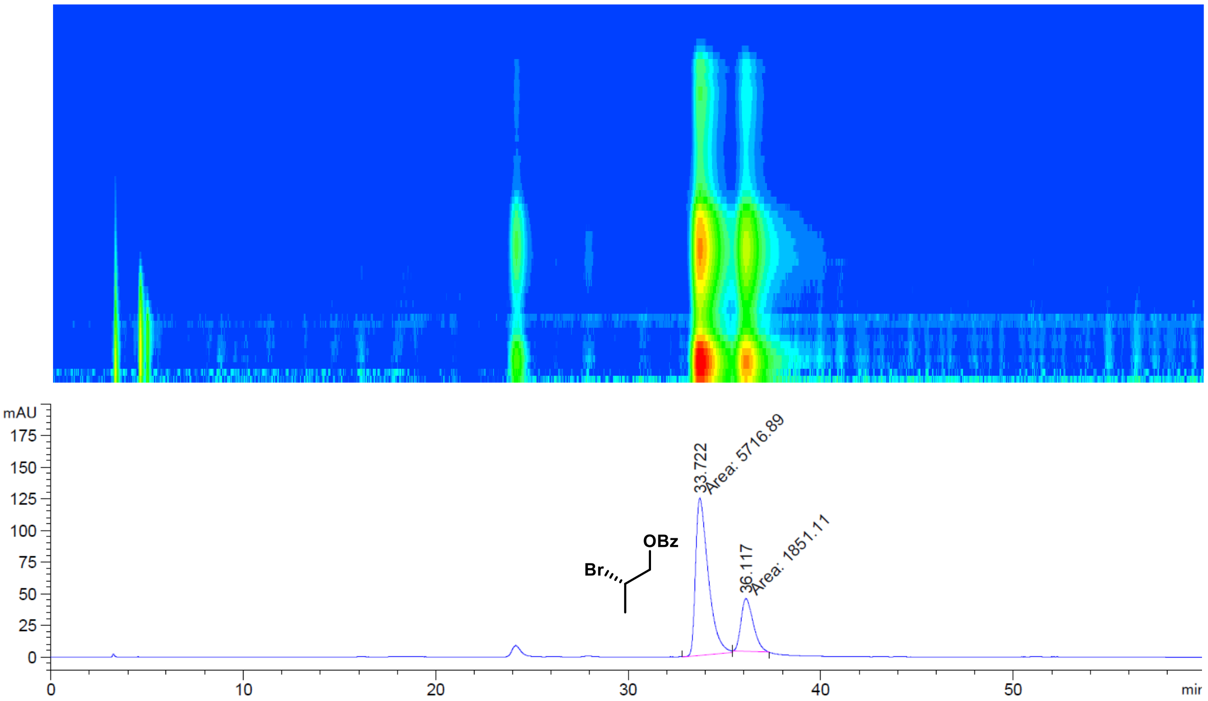

Peak #	RetTime [min]	Type	Width [min]	Area [mAU*s]	Height [mAU]	Area %
1	33.722	MM	0.7671	5716.89258	124.20947	75.5403
2	36.117	MM	0.7394	1851.11328	41.72502	24.4597

Chiralpak OD-H, 4.6 x 250 mm, 230 nm detection, 1 mL/min Hexane:IPA 99.5:0.5

2-Bromo-3-methylbutan-1-ol

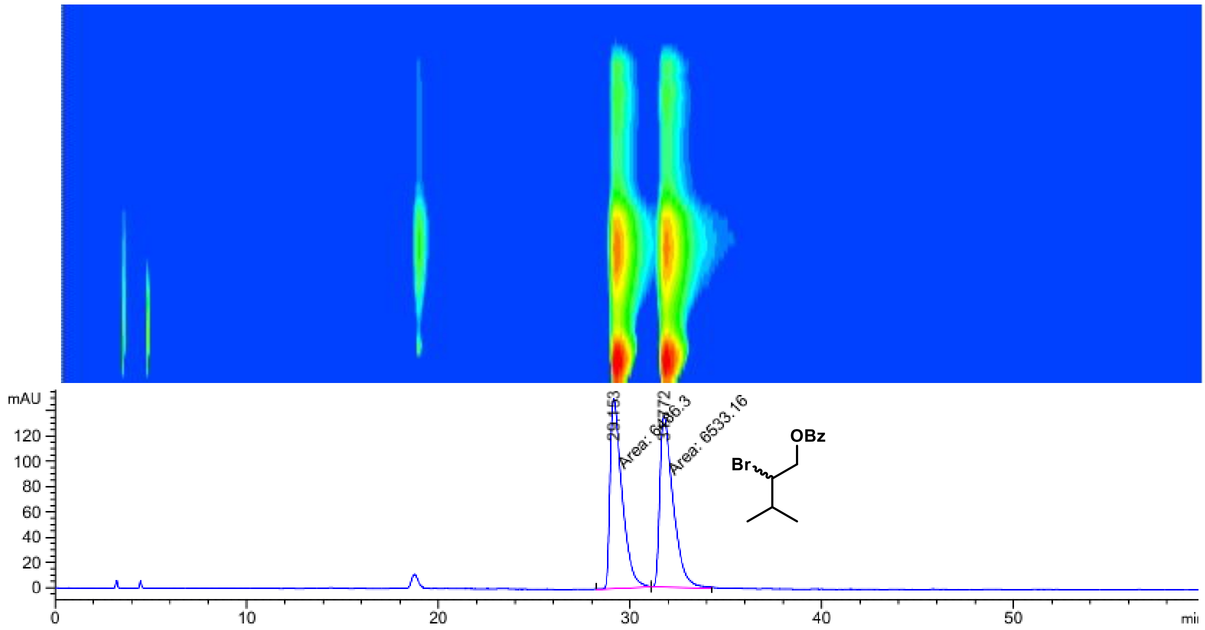

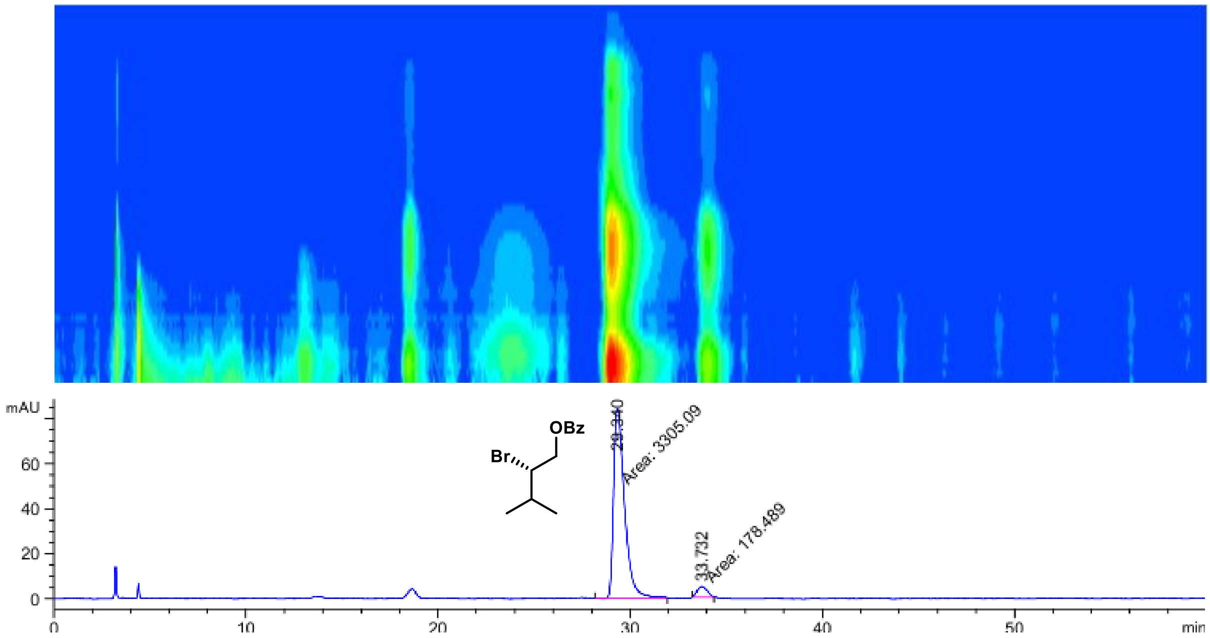

Peak #	RetTime [min]	Type	Width [min]	Area [mAU*s]	Height [mAU]	Area %
1	29.340	MM	0.6551	3305.08838	84.08279	94.8763
2	33.732	MM	0.6162	178.48932	4.82766	5.1237

Chiralpak OD-H, 4.6 x 250 mm, 220 nm detection, 1 mL/min Hexane:IPA 99:1

2-Bromo-3-cyclohexylpropylbenzoate

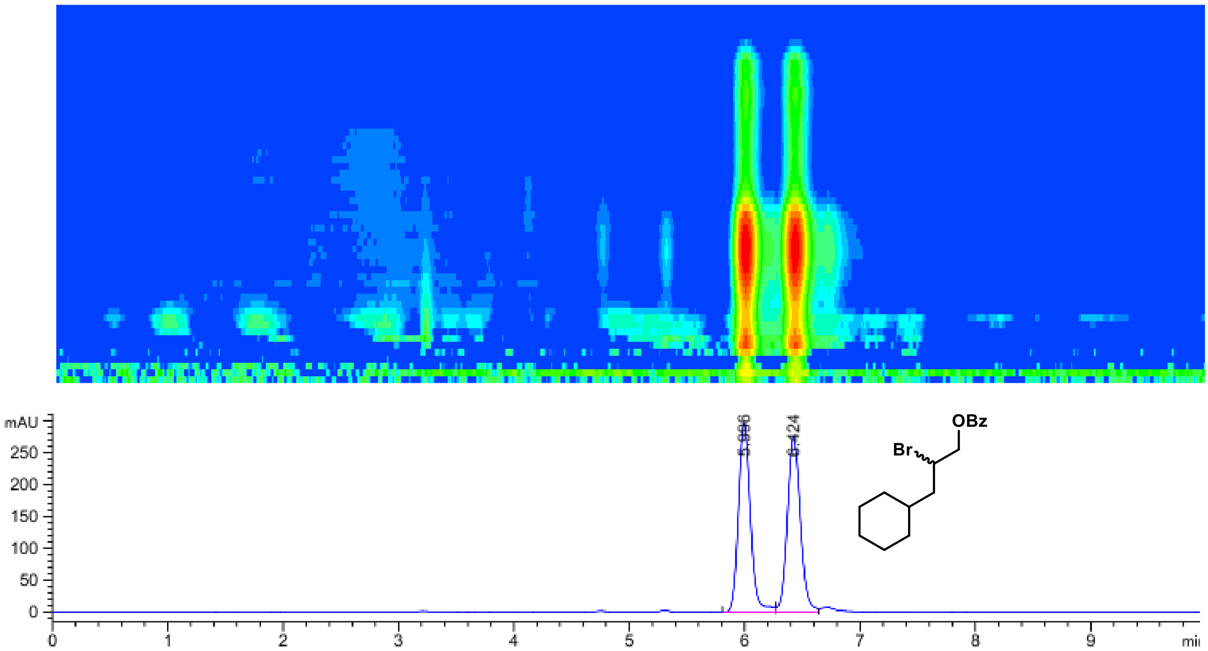

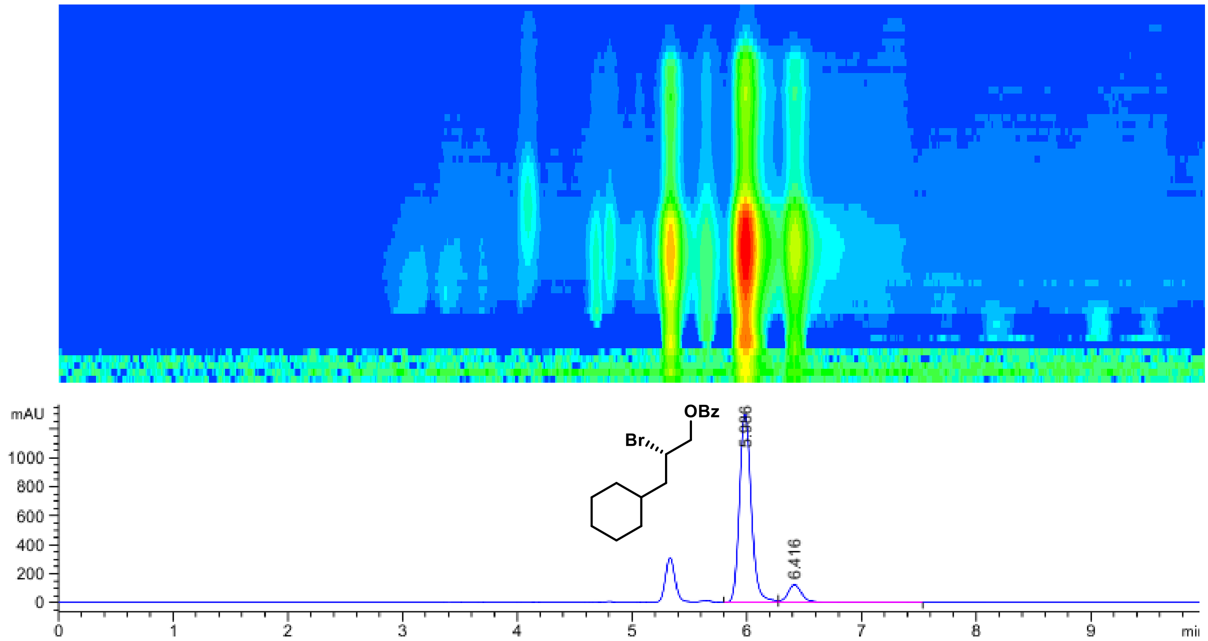

Peak #	RetTime [min]	Type	Width [min]	Area [mAU*s]	Height [mAU]	Area %
1	5.986	BV	0.1126	9421.87598	1299.27637	89.8180
2	6.416	VB	0.1285	1068.08887	124.04996	10.1820

Chiralpak OD-H, 4.6 x 250 mm, 230 nm detection, 1 mL/min Hexane:IPA 98.5:1.5

8. References

1. Hutchinson, G.; Alamillo-Ferrer, C.; Burés, J. *J. Am. Chem. Soc.* **2021**, *143*, 6805.

2. Grünenfelder, C. E.; Kisunzu, J. K.; Wennemers, H. *Angew. Chem., Int. Ed.* **2016**, *55*, 8571.

3. Takeshima, A.; Shimogaki, M.; Kano, T.; Maruoka, K. *ACS Catal*. **2020**, *10*, 5959.

4. Braddock, D. C.; Hermitage, S. A.; Kwok, L.; Pouwer, R.; Redmond, J. M.; White, A. J. P. *Chem. Commun.* **2009**, 1082.

5. Kano, T.; Shirozu, F.; Maruoka, K. *Chem. Commun*. **2010**, *46*, 7590.

6. Soroka, M.; Goldeman, W.; Maysa, P.; Stochaj, M. *Synthesis* **2003**, *15*, 2341.

7. Arnold, A. M.; Pöthig, A.; Drees, M.; Gulder, T. *J. Am. Chem. Soc.* **2018**, *140*, 4344.
